# Three New Ring-A Modified Ursane Triterpenes from *Davidia involucrata*

**DOI:** 10.3390/molecules19044897

**Published:** 2014-04-17

**Authors:** Qing-Wei Tan, Ming-An Ouyang, Bo Gao

**Affiliations:** 1 Key Laboratory of Bio-pesticide and Chemistry-Biology, Ministry of Education, Fujian Agriculture and Forestry University, Fuzhou 350002, Fujian, China; E-Mail: hotony@163.com; 2 Key Laboratory of Plant Virology of Fujian Province, Institute of Plant Virology, Fujian Agriculture and Forestry University, Fuzhou 350002, Fujian, China; 3 Fujian International Travel Health Care Center, Fuzhou 350001, Fujian, China; E-Mail: gaobo28083@sina.com; 4 Key Laboratory of Integrated Pest Management for Fujian-Taiwan Crops, Ministry of Agriculture, Fujian Agriculture and Forestry University, Fuzhou 350002, Fujian, China

**Keywords:** *Davidia involucrata*, Nyssaceae, triterpenoids, cytotoxicity

## Abstract

Three new ursane triterpenes, 3α,19α-dihydroxy-2-nor-urs-12-en-23,28-dioic acid-23-methyl ester (**1**), 19α,23-dihydroxy-3-oxo-2-nor-urs-12-en-28-oic acid (**2**), and 2,3-seco-3-methoxy-3,19α,23-trihydroxy-urs-12-en-2-al-28-oic acid (**3**), were isolated from the MeOH extract of the branch barks of *Davidia involucrata*, together with six known compounds. Their structures were elucidated by means of various spectroscopic analyses. The isolated triterpenes provide important evolutionary and chemotaxonomic knowledge about the monotypic genus *Davidia*. Five of the identified compounds showed moderate cytotoxicities against the cell proliferation of SGC-7901, MCF-7, and BEL-7404 with IC_50_ range from 7.26 to 47.41 μM.

## 1. Introduction

**Figure 1:**
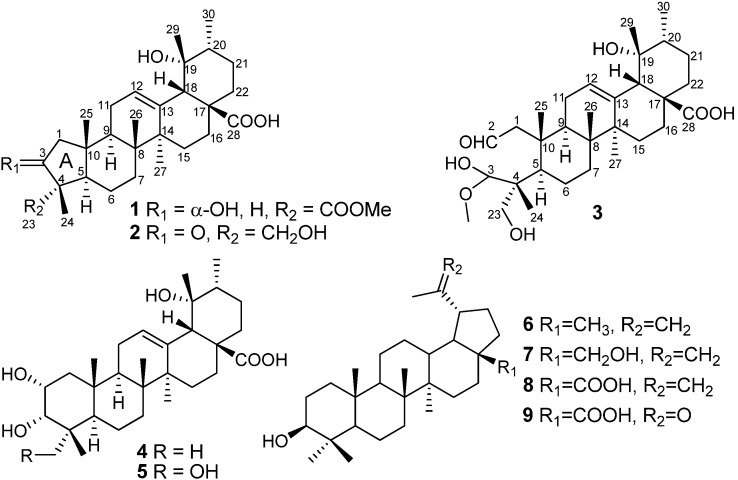
Compounds **1**–**9** isolated from the branch barks of *Davidia involucrata*.

*Davidia involucrata* Baill., the only species in the genus *Davidia* Baill., is a famous ornamental tree known as the Chinese dove tree or handkerchief tree. *D. involucrata* is a deciduous relic tree species of the Tertiary period with important ecological, scientific, and horticultural values [1,2,3]. More than 30 components, including flavonoids, sterols, triterpenes, alkaloids, and tannins, *etc.*' were identified from the aerial part of *D. involucrata* [4,5,6]. Our continuing efforts of the phytochemical study of this plant have led to the isolation of two new nor-ursane type triterpenoids with a five-membered A ring and a novel 2,3-seco-ursane triterpene, which were named davinvolunic acids A-C (compounds **1**–**3**). Moreover, two known ursane triterpenoids, euscaphic acid (**4**) [7] and myrianthic acid (**5**) [8], as well as four lupane triterpenes, including lupeol (**6**) [9], betulin (**7**) [10], betulinic acid (**8**) [11] and platanic acid (**9**) [12] were also obtained in the current study (Figure 1). This paper deals with the isolation and structure elucidation of the new compounds on the basis of spectroscopic methods, including 1D NMR, 2D NMR analyses, and MALDI-TOF-MS. Furthermore, all of the isolated triterpenoids except compound **2** were evaluated for their *in vitro* cytotoxic activities against three tumor cell lines (SGC-7901, MCF-7 and BEL-7404).

## 2. Results and Discussion

**Figure 2:**
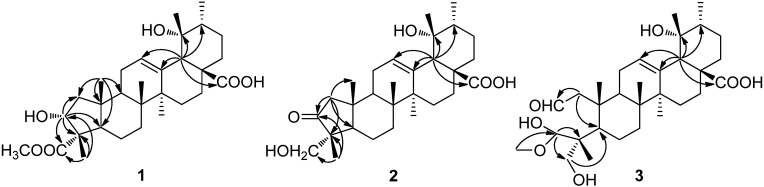
Key HMBC (H→C) correlations of compounds **1**–**3**.

Compound **1** was obtained as a white amorphous powder. The molecular formula, C_30_H_46_O_6_, which requires eight degrees of unsaturation, was determined on the basis of the [M + Na]^+^ ion peak at *m/z* = 525.3187 (calcd. 525.3192) in the HR-TOF-MS. The IR spectrum exhibited the presence of hydroxyl (3,430 cm^−1^) and carbonyl (1,750 cm^−1^ and 1,728 cm^−1^) groups. The ^13^C-NMR (125 MHz, CDCl_3_ and CD_3_OD) spectrum, together with DEPT and HSQC data, showed that three of the eight degrees of unsaturation of **1** came from one trisubstituted double bond at *δ*_C_ 128.7 and 138.5, and two carboxyl groups at *δ*_C_ 179.0 and 180.8. And the remaining five degrees of unsaturation were therefore indicative of the pentacyclic skeleton for **1**. In addition, three oxygenated carbons including a methyl (*δ*_C_ 51.9), a quaternary (*δ*_C_ 72.9) and a tertiary (*δ*_C_ 74.7) carbon were recognized in the ^13^C-NMR spectrum. Observed in the ^1^H-NMR (500 MHz, CDCl_3_ and CD_3_OD) spectrum were signals for five tertiary methyls at *δ*_H_ 0.62, 0.69, 1.15, 1.16 and 1.22 (s, each 3 H), one secondary methyl at *δ*_H_ 0.88 (d, *J* = 6.6 Hz, 3 H), one methoxy at *δ*_H_ 3.60 (s, 3 H), one oxymethine at *δ*_H_ 4.90 (t, *J* = 7.8 Hz, 1 H), and one olefinic proton at *δ*_H_ 5.27 (br.s, 1 H). The occurrence of a singlet signal at *δ*_H_ 2.47 (s, 1 H) for H-18 as well as a characteristic double of triplet signal at *δ*_H_ 2.42 (dt, *J* = 13.5, 5.0 Hz, 1 H) assignable to H-16α, which is caused downfield shift by the anisotropic effect due to a 19α-hydroxyl group, suggested that compound **1** is an urs-12-ene derivative possessing an α-hydroxyl group at C-19 [13,14]. The characteristic NMR data of compound **1** were comparable to those of euscaphic acid (**4**) [7] and myrianthic acid (**5**) [8], two known 19α-hydroxy-urs-12-en triterpenes that were also identified in the present investigation. Comparison of the 1D and 2D NMR data of **1** with those of **4** and **5** revealed that they shared the same B/C/D/E rings, and the only differences occurred in ring A. The unusual structure of a five-membered ring A was established by 2D NMR (HMBC and HSQC) experiments, based on the key HMBC correlations (Figure 2) observed from H_3_-25 (*δ*_H_ 0.62, s, 3 H) to C-1, C-5, C-9 and C-10, from H-3 (*δ*_H_ 4.90, t, *J* = 7.8 Hz, 1 H) to C-1, C-4, C-5 and C-23, from H-1a (*δ*_H_ 2.09, dd, *J* = 11.5, 7.5 Hz, 1 H) to C-3, C-4, C-5 and C-25, and from H_3_-24 (*δ*_H_ 1.16, s, 3 H) to C-3, C-4 and C-23. The relative stereochemistry of **1** was established by the ROESY spectrum. The observed cross-peaks of H-3/H_3_-24, H_3_-25, H_3_-26 and H-18/H_3_-29 implied that they were cofacial and placed in β-orientation, and therefor indicated the α-orientation of the hydroxyl group at C-3. The ROESY correlations of H_3_-23/H-5, H-5/H-9 and H-9/H-27 revealed that they had α-configurations. Compound **1** was therefore determined as 3α,19α-dihydroxy-2-nor-urs-12-en-23,28-dioic acid-23-methyl ester, and named davinvolunic acid A.

Compound **2** was obtained as a white amorphous powder. Its molecular formula, C_29_H_44_O_5_, was deduced from its HR-TOF-MS [M + Na]^+^ ion at *m/z* = 495. 3080 (calcd. 495.3086), indicating eight degrees of unsaturation. The IR spectrum revealed the presence of hydroxyl (3,428 cm^−1^) and carbonyl (1,686 cm^−1^ and 1,740 cm^−1^). The NMR spectra of **2** were similar to those of **1**, with the differences only occurred in ring A due to different substituents. When comparing the ^13^C-NMR spectra of these two compounds, the signals of a COOMe group at *δ*_C_ 179.0 and 51.9, and an oxygenated tertiary carbon at *δ*_C_ 74.7 (C-3) in compound **1** were absent, while an oxygenated secondary carbon at *δ*_C_ 67.8 and a keto group at *δ*_C_ 225.4 appeared instead. The keto group was positioned at C-3, while an hydroxyl was attached to C-23, which was confirmed by the observed HMBC correlations (Figure 2) from H_3_-24 (*δ*_H_ 0.91, s, 3 H) to C-3, C-4 and C-23, and the key HMBC correlations from H-1 (*δ*_H_ 2.07, s, 2 H) to C-3, C-4, C-5 and C-25.

In comparison to the common keto carbonyl group of six-membered ring A analogues [15,16], the chemical shift of C-3 was notably downfield shifted due to the higher strain of the contracted five-membered ring A [17]. Further analysis of the NOESY experiment revealed the consistent relative configuration of **2** with that of **1**. Thus, the structure of compound **2**was deduced as 19α,23-dihydroxy-3-oxo-2-nor-urs-12-en-28-oic acid, and named as davinvolunic acid B.

Compound **3**, obtained as a white amorphous powder, had the molecular formula C_31_H_50_O_7_, which was established by HR-TOF-MS [M + Na]^+^ ion at *m/z* = 557.3448 (calcd. 5557.3454), with one degree of unsaturation less than that of compounds **1** and **2**. The IR spectrum exhibited absorption bands for hydroxyl (3,437 cm^−1^) and carbonyl (1,741 cm^−1^ and 1,720 cm^−1^) groups. The ^1^H-NMR (500 MHz, CDCl_3_ and CD_3_OD) spectrum showed the presence of a secondary methyl group at *δ*_H_ 0.83 (d, *J* = 6.7 Hz, 3 H), an olefinic proton at *δ*_H_ 5.26 (br. s, 1 H), a sharp singlet at *δ*_H_ 2.45 (s, 1 H), and a double of triplet signal at *δ*_H_ 2.37 (dt, *J* = 13.5, 5.0 Hz, 1 H). These characteristic data indicated that **3** also had a 19α-OH substituted urs-12-ene type skeleton. The ^13^C-NMR (100 MHz, CDCl_3 _and CD_3_OD) spectrum with DEPT experiments resolved 31 carbon resonances and indicated that three of the seven degree of unsaturation came from one double bond, one carbonyl, and one aldehyde group, while the remaining four degree of unsaturation suggested that compound **3** might be a tetracyclic triterpene. Careful comparison of the ^1^H-NMR and ^13^C-NMR data of **3** with those of compound **1** and **2** revealed that they shared the same B/C/D/E rings. The HSQC, HMBC, and NOESY 2D-NMR spectra turned out to be much more complex, resulted from the change of the structure of **3** due to the unstable substituted situation at C-3 as presented. Fortunately, the key HMBC correlations (Figure 2) from H-3 (*δ*_H_ 4.45, dd, *J* = 9.5, 5.0 Hz) to C-4 (*δ*_C_ 53.2) and C-23 (*δ*_C_ 65.0), from H-23a (*δ*_H_ 3.60, d, *J* = 13.2 Hz, 1H) and H-23b (*δ*_H_ 3.69, d, *J* = 13.2 Hz, 1H) to C-3 (*δ*_C_ 100.3), C-5 (*δ*_C_ 60.7), and from H-1a (*δ*_H_ 2.03, m) to C-2 (*δ*_C_ 206.6) and C-5 (*δ*_C_ 60.7) could still be recognized.

**Figure 3:**
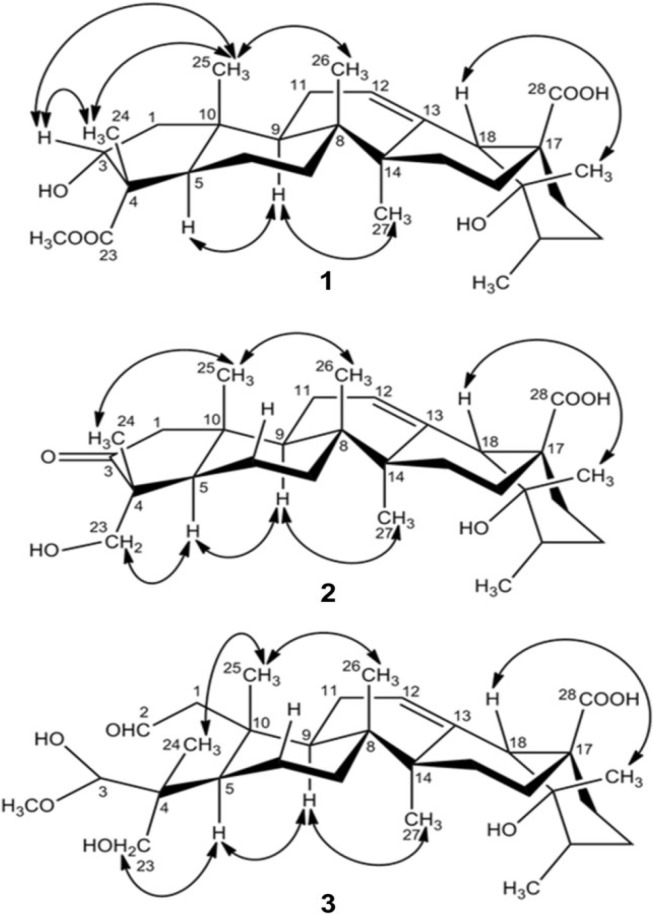
Key ROESY correlations of compounds **1–3**.

Analysis of the ROESY spectrum (Figure 3) suggested the relative configuration of the remainder of the molecule of **3** was identical to those of **1** and **2**. Compound **3** was thus deduced as 2,3-seco-3-methoxy-3,19α,23-trihydroxy-urs-12-en-2-al-28-oic acid, and named davinvolunic acid C.

Davinvolunic acids A (**1**) and B (**2**) are novel nor-triterpenes having a five-membered A-ring, while davinvolunic acid C (**3**) appears to be a 2,3-seco triterpene. They are all 12-en-ursane triterpenoids bearing an α-hydroxyl group at C-19 and a carboxyl group at C-17, the same as with euscaphic acid (**4**) and myrianthic acid (**5**). The compounds reported in the present study, especially the three novel and unique structures, which reflects to some extent the structural diversity of the triterpenes from *D. involucrata*, provide important evolutionary and chemotaxonomic knowledge of the monotypic genus *Davidia*. The cytotoxic activities of the isolated triterpenoids were determined using MTT method against SGC-7901, MCF-7 and BEL-7404, with doxorubicin as positive control. The IC_50_ value was defined as the concentration of the test compounds necessary to inhibit the growth to 50% of the control in the MTT assay. The cytotoxic activities of these compounds against human cell lines *in vitro* were summarized in Table 1. Among the tested compounds, **3** and **6**–**9** showed moderate cytotoxicity against all tumor cells, while compound **8** exhibited the most potent cytotoxicity with IC_50_ value of 7.26–12.37 μM.

**Table 1:** Cytotoxicity of compounds **1**–**9** against three human tumor cell lines (IC_50_, μM).

Compounds	Cell lines		
	SGC-7901	MCF-7	BEL-7404
**1**	– ^a^	–	–
**2**	Nt	Nt	Nt
**3**	36.3 ± 2.00	37.4 ± 1.57	47.41 ± 20.4
**4**	–	–	–
**5**	–	–	–
**6**	42.35 ± 1.79	45.8 ± 2.15	38.24 ± 1.57
**7**	39.25 ± 1.83	21.24 ± 1.15	24.59 ± 1.51
**8**	7.26 ± 0.08	12.37 ± 0.18	8.15 ± 0.13
**9**	10.26 ± 0.52	15.21 ± 0.28	18.14 ± 0.30
Doxorubicin	0.19 ± 0.032	0.08 ± 0.007	0.12 ± 0.011

Nt: not tested; ^a^ The IC_50_ values for cytotoxicity of compounds against cells were given when IC_50_ were less than 100 μM.

Triterpenoid derivatives have been one of the most interesting areas of research in the past few years due to their broad range of biological and medicinal properties [18,19,20]. Davinvolunic acid A (**1**), euscaphic acid (**4**), and myrianthic acid (**5**), with an α-oriented hydroxyl group at C-3, were found to show no cytotoxicity against all the three tumor cell lines tested at 100 μM. The experimental results were consistent with a SAR study in the literature [21], which indicated that ursolic acid derivatives possessing two hydrogen-bond forming groups (an H-donor and a carbonyl group) at positions 3 and 28 exhibit cytotoxic activity, and compounds with a β-oriented hydrogen-bond forming groups at C-3 exhibit more potent cytotoxicity than the α-counterparts.

Compounds **6**–**9** belong to the pentacyclic lupane-type triterpene class. Betulinic acid (**8**) and platanic acid (**9**), with a hydroxyl group at C-17, were found to show higher cytotoxic activity than lupeol (**6**) and betulin (**7**). Betulinic acid (**8**), one of such compounds that possess several medicinal properties including anticancer, antimalarial, antimicrobial and anti-HIV activities, was initially considered to be melanoma-specific, but recent studies suggest that it shows anticancer activity against a broad panel of cancers [22,23,24,25].

## 3. Experimental Section

### 3.1. General

Optical rotations were measured with a Jasco DIP-180 digital polarimeter. IR spectra were recorded with a Perkin-Elmer 1750 FT-IR spectrometer in KBr discs. High-resolution mass spectra were recorded on an IonSpec 4.7 Tesla FTMS instrument. All NMR spectra were obtained using a Bruker AV-400 or a DRX-500 spectrometer. Semipreparative HPLC was performed with an Elite P230 pump equipped with a Schambeck SFD GmbH RI2000 detector and a YMC-Pack SIL column (250 × 10 mm, 5 µm). Sephadex LH-20 (25–100 µm, Pharmacia Fine Chemicals, Uppsala, Sweden), Silica gel (200–300 mesh) and Silica gel H (Qingdao Oceanic Chemical Co., Qingdao, China) were used for column chromatography. TLC was performed on 0.25 mm thick silica prepared plates (H, Qingdao Oceanic Chemical Co.) using the solvent system CH_3_Cl/MeOH (100/5), with spot visualization by spraying with 5% (v/v) H_2_SO_4_ in alcohol solution followed by heating.

### 3.2. Plant Material

The branch barks of *D. involucrata* were collected from Shennongjia Forest Region of Hubei Province, China in 2002. The plant was authenticated by Prof. Y.P. Yang. A voucher specimen (No. 12245) is deposited in the Herbarium of Kunming Institute of Botany, Chinese Academy of Sciences, Kunming, China.

### 3.3. Extraction and Isolation

The air-dried branch barks of the plant (10 kg) were extracted with MeOH (2 × 10 L) at room temperature to give 600 g of crude extract, which was solubilized in water (1 L) and then filtered. The water-insoluble fraction (175 g) was subjected to a silica gel column (200–300 mesh, petroleum ether/acetone, 10:1 to 10:5) to give seven fractions 1–7.

Fraction 5 (6 g) was subjected to Sephadex LH-20 column chromatography eluted with CHCl_3_/MeOH (1:1) to afford five fractions 5-1–5-5. Fraction 5-2 was subjected to silica gel column (petroleum ether/CHCl_3_/acetone, 10:6:2) to give **6** (12.6 mg) and **7** (650.0 mg). Fraction 5-5 was obtained as white powder (**8**, 21.3 mg).

Fraction 6 (9 g) was subjected to Sephadex LH-20 column chromatography eluted with MeOH to afford five fractions 6-1–6-5. Fraction 6-3 was purified by silica gel H column chromatography (CHCl_3_/MeOH, 100:1 to 100:2) to afford **3** (12.3 mg) and **9** (11.1 mg). Fraction 6-4 was subjected to silica gel H column chromatography (CHCl_3_/MeOH, 100:1 to 100:3), and then purified by semi-preparative HPLC (CHCl_3_/MeOH, 100:3) to give **1** (11.7 mg) and **2** (5.1 mg).

Fraction 7 (8 g) was subjected to Sephadex LH-20 column eluted with MeOH to afford four fractions 7-1–7-4. Fraction 7-3 was purified by semi-preparative HPLC (CHCl_3_/MeOH, 100:4) to give **4** (19.9 mg) and **5** (8.5 mg).

**Table 2:** NMR data for compound **1**–**3**.

No.	1 ^a^		2 ^b^		3 ^b^	
	*δ*_C_ (DEPT)	*δ*_H_, mult. (*J* in Hz)	*δ*_C_ (DEPT)	*δ*_H_, mult. (*J* in Hz)	*δ*_C_ (DEPT)	*δ*_H_, mult. (*J* in Hz)
1	50.3 (CH_2_)	0.97 d (11.5), 2.09 dd (11.5, 7.5)	56.5 (CH_2_)	2.07 s	43.9 (CH_2_)	1.30 m, 2.03 m
2	–	–	–	–	206.6 (CH)	9.85 s
3	74.7 (CH)	4.90 t (7.8)	225.4 (C)	–	100.3 (CH)	4.45 dd (9.5, 5.0)
4	53.1 (C)	–	51.4 (C)	–	53.2 (C)	–
5	61.4 (CH)	1.19 m	51.4 (CH)	1.65 m	60.7 (CH)	0.94 m
6	20.8 (CH_2_)	1.44 m, 1.70 m	18.7 (CH_2_)	1.49 m, 1.77 m	20.3 (CH_2_)	1.58 m, 1.85 m
7	33.3 (CH_2_)	1.19 m, 1.50 m	32.5 (CH_2_)	1.22 m, 1.53 m	33.2 (CH_2_)	1.32 m, 1.48 m
8	40.2 (C)	–	40.2 (C)	–	39.9 (C)	–
9	45.7 (CH)	1.71 m	44.9 (CH)	1.58 m	43.1 (CH)	1.54 m
10	41.1 (C)	–	40.6 (C)	–	40.1 (C)	–
11	25.4 (CH_2_)	1.88 m, 1.92 m	25.4 (CH_2_)	1.86 br. d (15.8), 2.18 br. d (15.8)	24.7 (CH_2_)	1.89 m, 2.02 m
12	128.7 (CH)	5.27 br. S	128.2 (CH)	5.32 br. s	128.8 (CH)	5.26 br. s
13	138.5 (C)	–	138.8 (C)	–	138.0 (C)	–
14	41.7 (C)	–	41.7 (C)	–	41.4 (C)	–
15	28.2 (CH_2_)	0.95 m, 1.67 m	28.1 (CH_2_)	0.97 m, 1.64 m	28.0 (CH_2_)	0.94 m, 1.61 m
16	25.4 (CH_2_)	1.50 m, 2.42 dt (13.5, 5.0)	25.5 (CH_2_)	2.49 dt (13.5, 5.0)	25.3 (CH_2_)	2.37 dt (13.5, 5.0)
17	47.6 (C)	–	47.5 (C)	–	47.9 (C)	–
18	53.4 (CH)	2.47 s	53.3 (CH)	2.53 s	53.2 (CH)	2.45 s
19	72.9 (C)	–	72.9 (C)	–	72.9 (CH_3_)	–
20	41.1 (CH)	1.20 m	41.1 (CH)	1.22 m	41.0 (C)	1.29 m
21	25.9 (CH_2_)	1.58 m, 1.73 m	25.9 (CH_2_)	1.59 m, 1.78 m	25.8 (CH)	1.56 m, 1.80 m
22	37.4 (CH_2_)	1.45 m, 1.69 m	37.4 (CH_2_)	1.50 m, 1.68 m	37.2 (CH_2_)	1.55 m, 1.60 m
23	179.0 (CH_3_)	–	67.8 (CH_2_)	3.26 (10.7), 3.66 d (10.7)	65.0 (CH_2_)	3.60 (13.2), 3.69 d (13.2)
24	21.0 (CH_3_)	1.16 s	17.6 (CH_3_)	0.91 s	20.2 (CH_3_)	0.88 s
25	15.3 (CH_3_)	0.62 s	16.0 (CH_3_)	0.91 s	14.0 (CH_3_)	0.96 s
26	16.5 (CH_3_)	0.69 s	16.9 (CH_3_)	0.81 s	17.0 (CH_3_)	0.75 s
27	24.5 (CH_3_)	1.22 s	24.8 (CH_3_)	1.33 s	23.7 (CH_3_)	1.16 s
28	180.8 (C)	–	180.8 (C)	–	180.7 (C)	–
29	27.0 (CH_3_)	1.15 s	27.1 (CH_3_)	1.18 s	26.7 (CH_3_)	1.09 s
30	15.9 (CH_3_)	0.88 d (6.6)	16.6 (CH_3_)	0.92 d (6.0)	15.8 (CH_3_)	0.83 d (6.7)
OMe	51.9 (CH_3_)	3.60 s	–	–	54.4 (CH_3_)	3.22 s

^a^: Recorded in CDCl_3_ and CD_3_OD (10:1), ^1^H-NMR in 500 MHz, while ^13^C-NMR in 125 MHz, *δ* in ppm. ^b^: Recorded in CDCl_3_ and CD_3_OD (10:1), ^1^H-NMR in 500 MHz, while ^13^C-NMR in 100 MHz, *δ* in ppm.

### 3.4. Spectral Data

*Davinvolunic acid A* (**1**). White amorphous powder. 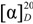 = +87.5 (c 0.12, CHCl_3_). IR (KBr) ν_max_ (cm^−1^): 3430, 2950, 1750, 1728. HR-TOF-MS *m/z* = 525.3187 [M + Na]^+^ (calcd. for C_30_H_46_O_6_Na^+^ 525.3192). ^1^H-NMR (500 MHz, CDCl_3_ and CD_3_OD) and ^13^C-NMR (125 MHz, CDCl_3_ and CD_3_OD) data (see Table 2).

*Davinvolunic acid B* (**2**). White amorphous powder. 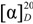 = +109.8 (c 0.22, CHCl_3_). IR (KBr) ν_max_ (cm^−1^): 3428, 2920, 1740, 1686. HR-TOF-MS *m/z* = 495.3080 [M + Na]^+^ (calcd. for C_29_H_44_O_5_Na 495.3086). ^1^H-NMR (500 MHz, CDCl_3_ and CD_3_OD) and ^13^C-NMR (100 MHz, CDCl_3_ and CD_3_OD) data (see Table 2).

*Davinvolunic acid C* (**3**). white amorphous powder. 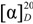 = +124.2 (c 0.15, CHCl_3_). IR (KBr) ν_max_ (cm^−1^): 3437, 2930, 1741, 1720. HR-TOF-MS *m/z* 557.3448 [M + Na]^+^ (calcd. for C_31_H_50_O_7_Na 557.3454). ^1^H-NMR (500 MHz, CDCl_3_ and CD_3_OD) and ^13^C-NMR (100 MHz, CDCl_3_ and CD_3_OD) data (see Table 2).

### 3.5. Cytotoxicity Assay

The *in vitro* cytotoxic activity was determined by the MTT colorimetric method. Three tumor cell lines, SGC-7901 cells (human gastric adenocarcinoma), MCF-7 cells (human breast cancer) and BEL-7404 (human hepatocellular carcinoma), provided by Department of Hepatobiliary Surgery, Affiliated Union Hospital, Fujian Medical University, were cultured in RPMI-1640 medium supplemented with 10% fetal bovine serum and 100 IU·mL^−1^ of penicillin-streptomycin at 37 °C in humidified atmosphere with 5% CO_2_. For the cytotoxicity tests, cells in exponential growth stage were harvested from culture by trypsin digestion and centrifuging at 180×g for 3 min, and then resuspended in fresh medium at a cell density of 5 × 10^4^ per ml. The cell suspension was dispensed into a 96-well microplate at 100 μL and incubated for 24 h, and then treated with compounds at various concentrations. After 48 h of treatment, 50 μL solution of 1 mg·mL^−1^ MTT was added to each well, and the cells were further incubated for 4 h. Finally, supernatants were removed and the formazan crystals were dissolved by adding 100 μL DMSO and the optical density was recorded at 570 nm. All drug doses were tested with doxorubicin as positive control in triplicate.

## 4. Conclusions

Chemical investigation of the branch barks of *D. involucrata* resulted in the isolation of two nor- and one seco-triterpenoids, namely 3α,19α-dihydroxy-2-nor-urs-12-en-23,28-dioic acid-23-methyl ester (**1**), 19α,23-dihydroxy-3-oxo-2-nor-urs-12-en-28-oic acid (**2**), and 2,3-seco-3-methoxy-3,19α,23-trihydroxy-urs-12-en-2-al-28-oic acid (**3**), together with six known compounds **4**–**9**. Five of the identified compounds showed moderate cytotoxicities against three tumor cell lines with IC_50_s ranging from 7.26 to 47.41 μM. A known lupane triterpene, betulinic acid (**8**), possesses the most significant inhibitory effect against the cell proliferation of SGC-7901, MCF-7, and BEL-7404 with IC_50_ 7.26, 12.37, and 8.15 μM.

## Supplementary Materials

Supplementary materials can be accessed at: http://www.mdpi.com/1420-3049/19/4/4897/s1.
