# Supplementary material for: Three New Ring-A Modified Ursane Triterpenes from Davidia involucrata
Source: Molecules. 2014 Apr 17;19(4):4897–906. doi: 10.3390/molecules19044897 (PMC6271727; doi:10.3390/molecules19044897)

Supporting Information

Figure S1. ^{1}H-NMR spectrum (500 MHz) of Davinvolunic acid A (1) in $CDCl_3$ and CD_3OD (10:1).

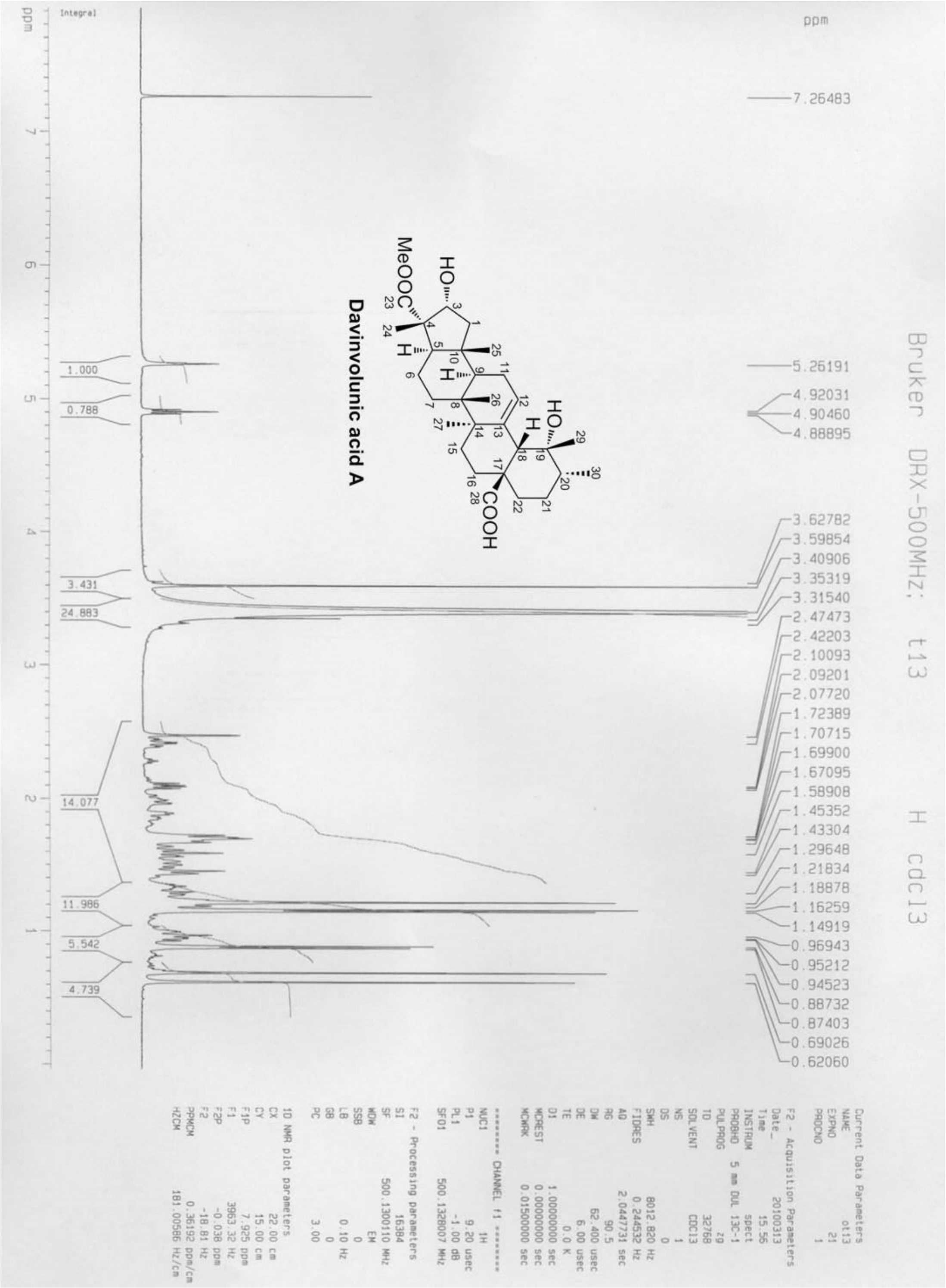

Figure S2. ^{13}C-NMR spectrum (125 MHz) of Davinvolunic acid A (1) in $CDCl_3$ and CD_3OD (10:1).

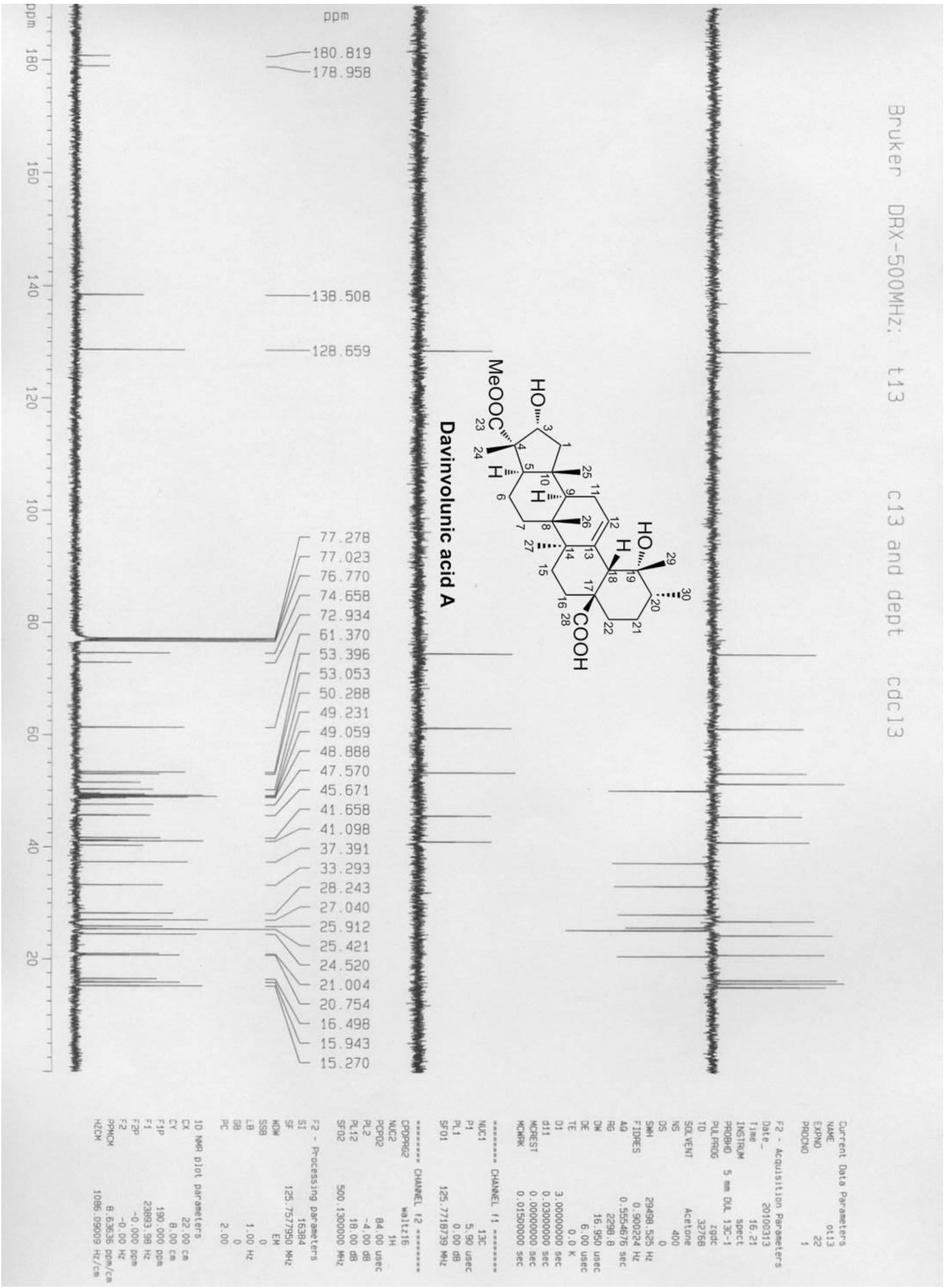

Figure S3. MALDI-TOF-MS spectrum of Davinvolunic acid A (1).

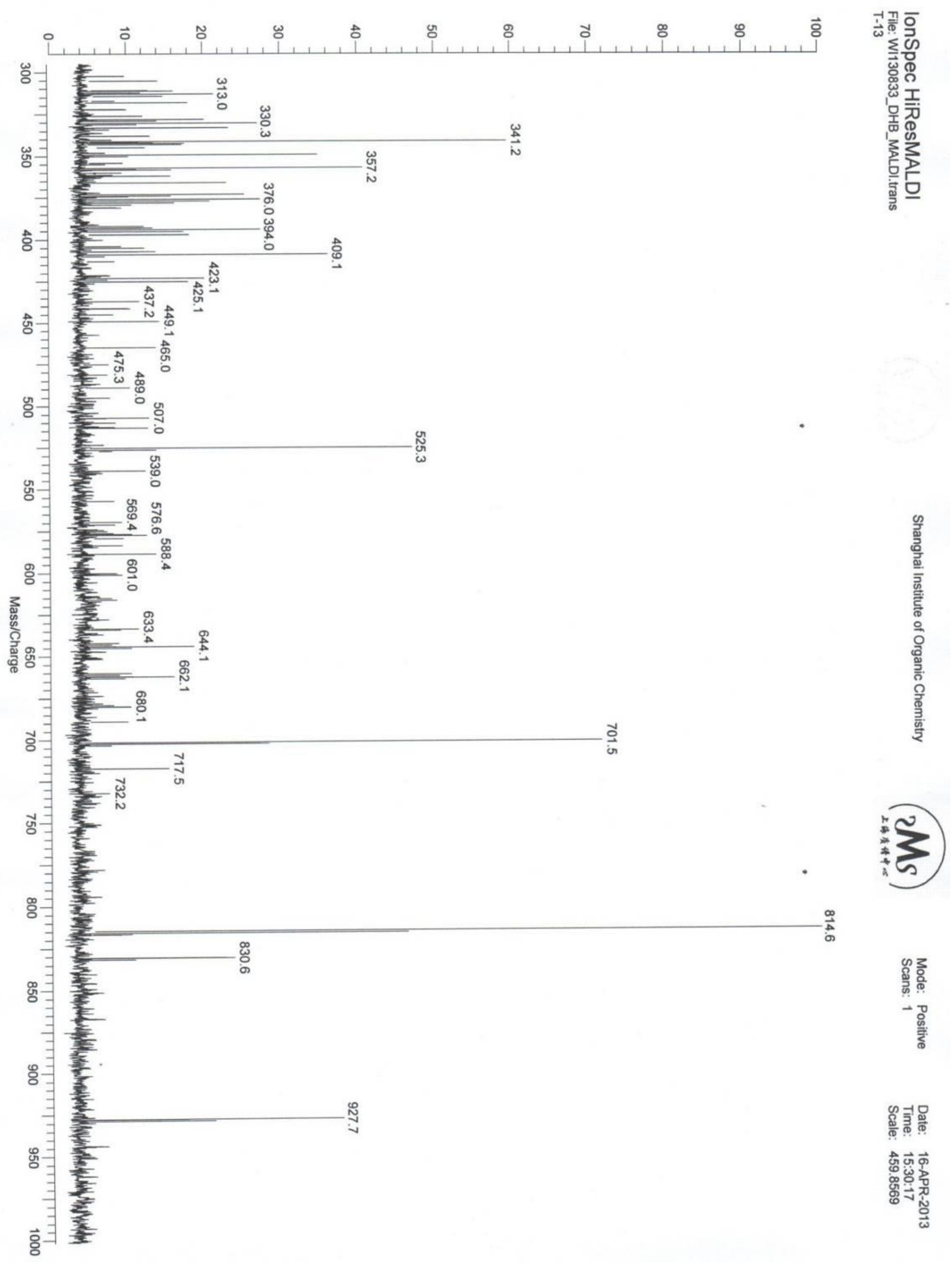

Figure S4. ^{1}H-NMR spectrum (500 MHz) of Davinvolunic acid B (2) in $CDCl_3$ and CD_3OD (10:1).

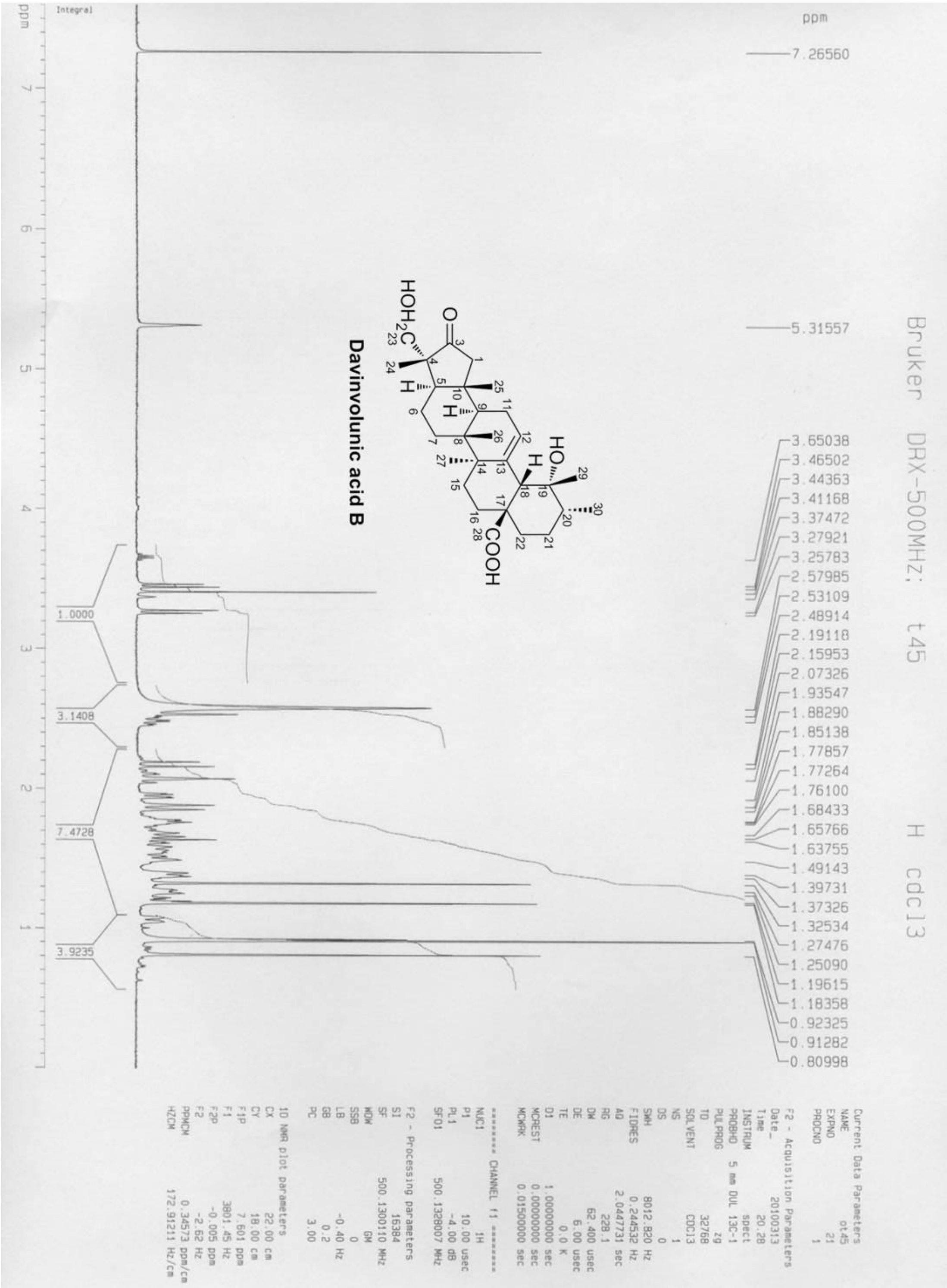

Figure S5. ^{13}C-NMR spectrum (100 MHz) of Davinvolunic acid B (2) in $CDCl_3$ and CD_3OD (10:1).

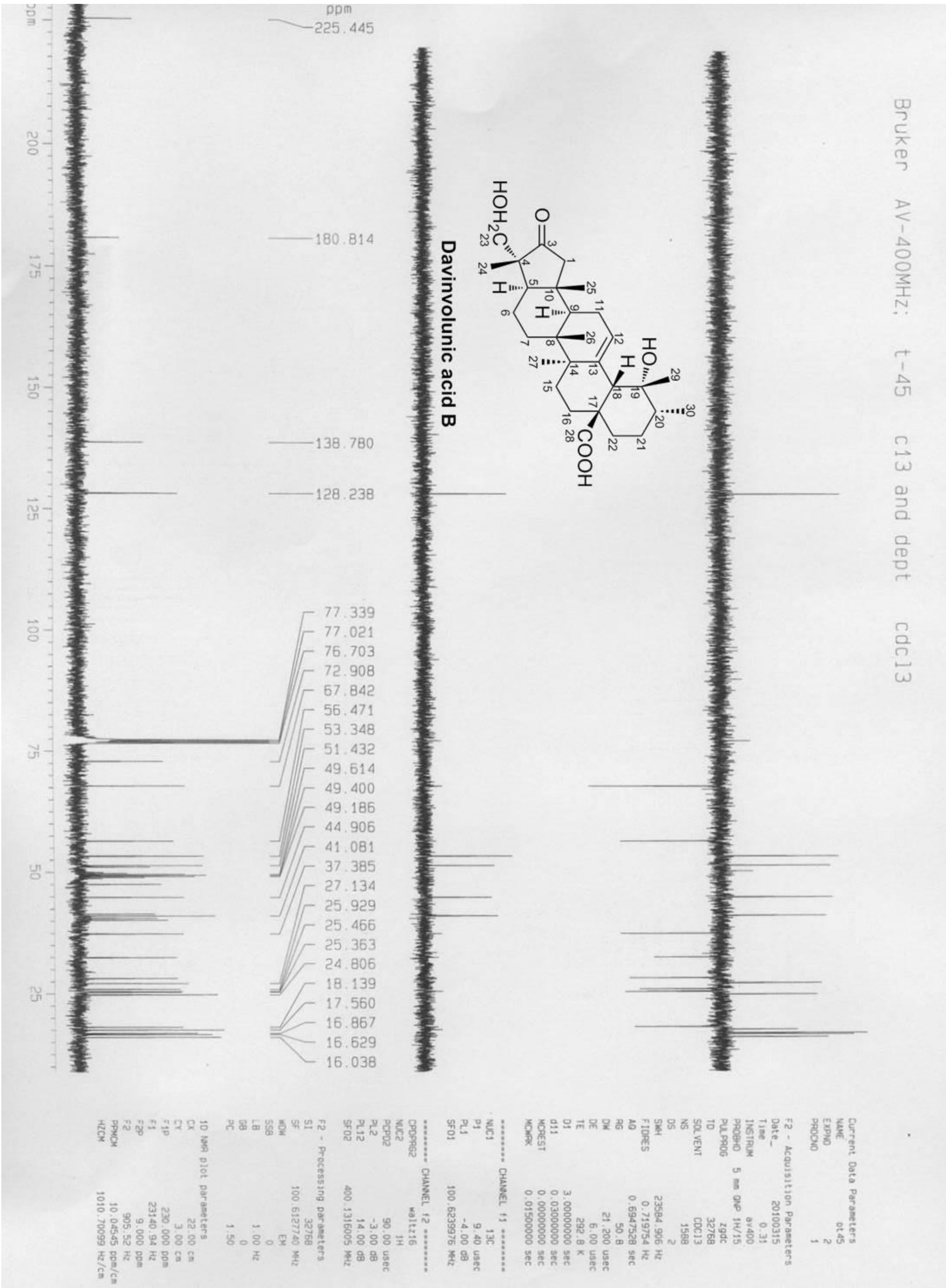

Figure S6. MALDI-TOF-MS spectrum of Davinvolunic acid B (2).

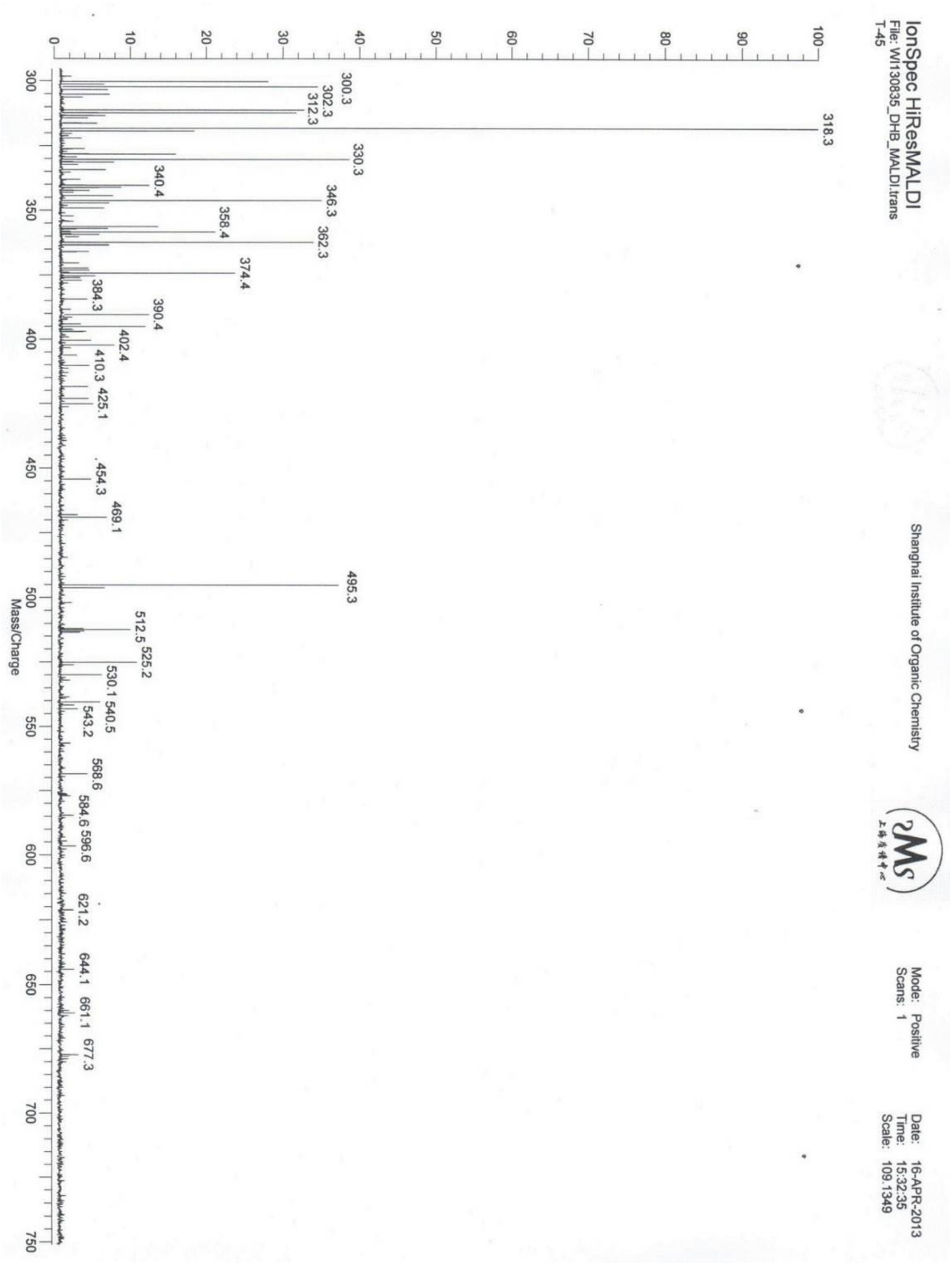

Figure S7. ^{1}H-NMR spectrum (400 MHz) of Davinvolunic acid C (3) in $CDCl_3$ and CD_3OD (10:1).

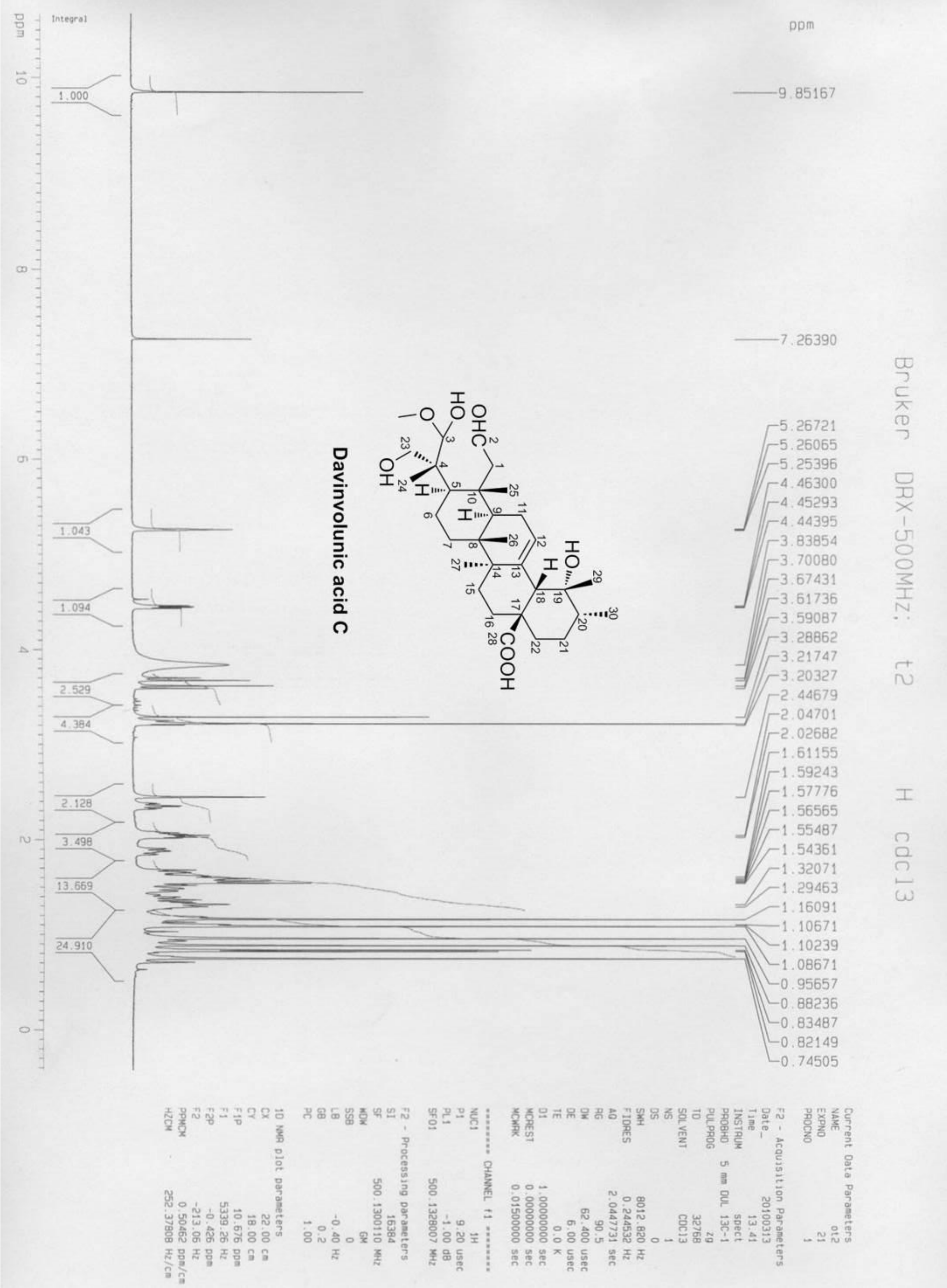

Figure S8. ^{13}C-NMR spectrum (100 MHz) of Davinvolunic acid C (3) in $CDCl_3$ and CD_3OD (10:1).

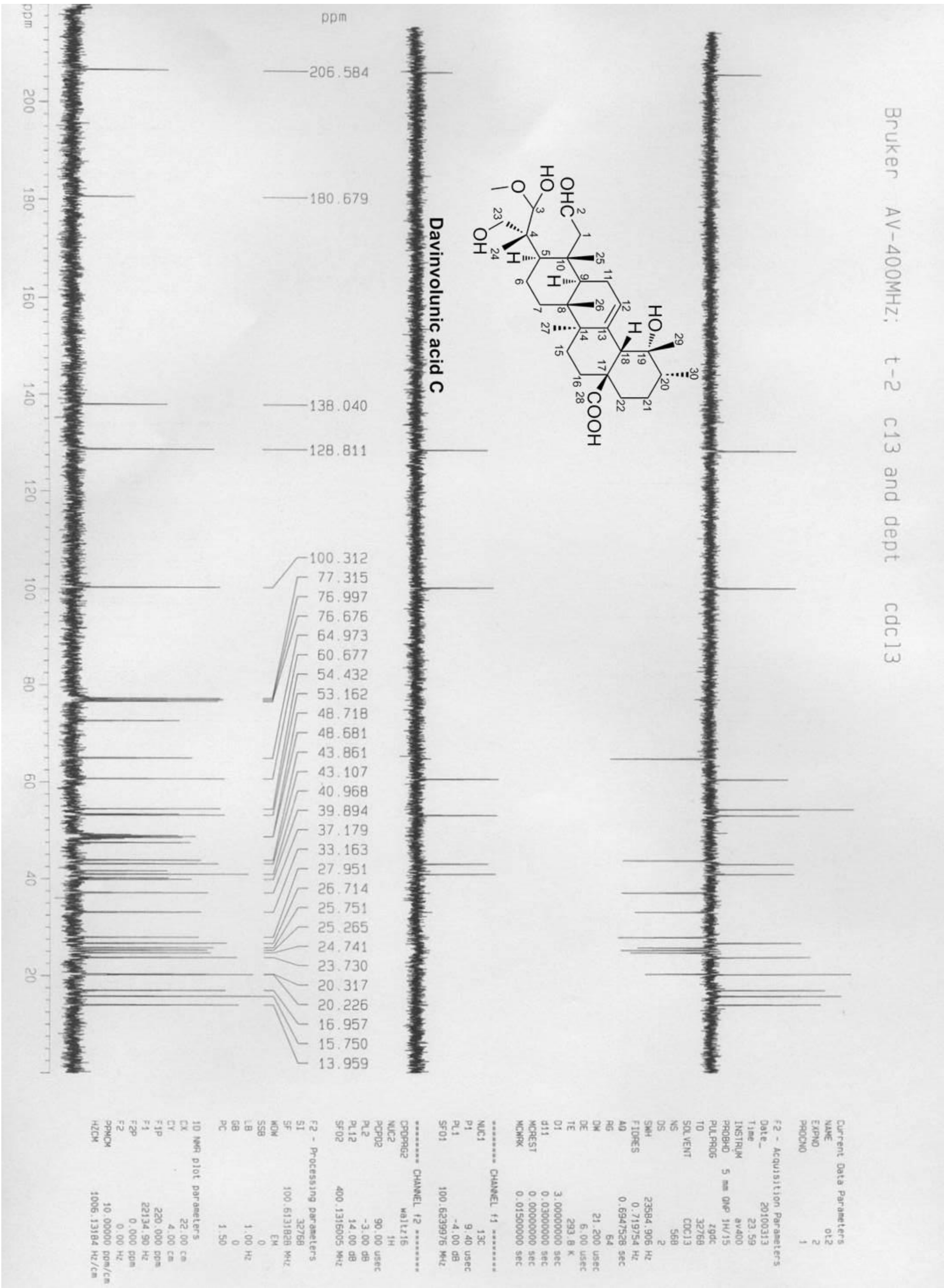

Figure S9. MALDI-TOF-MS spectrum of Davinvolunic acid C (3).

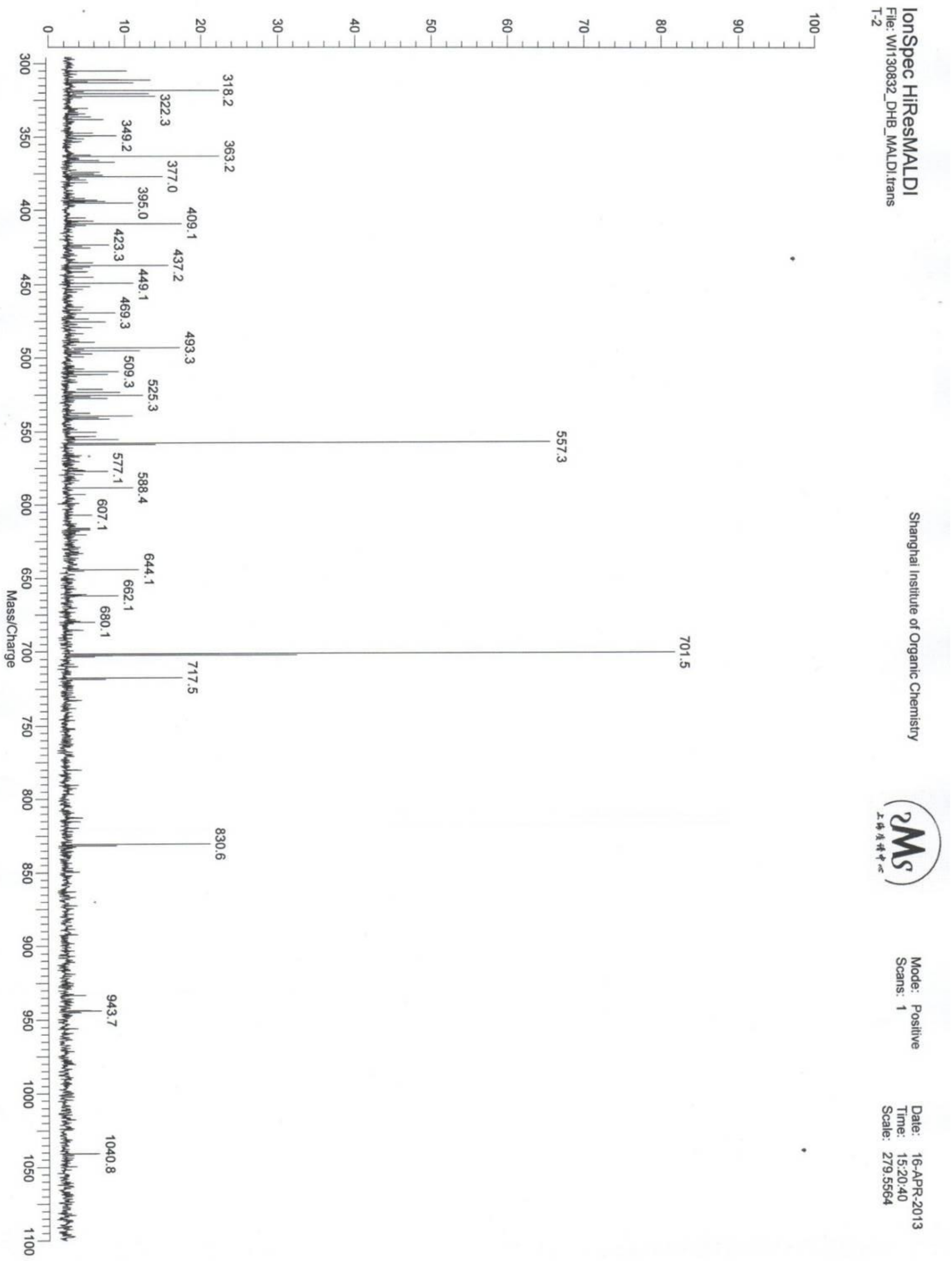

Figure S10. ^{1}H-NMR spectrum (500 MHz) of Euscaphic acid (4) in C_5D_5N.

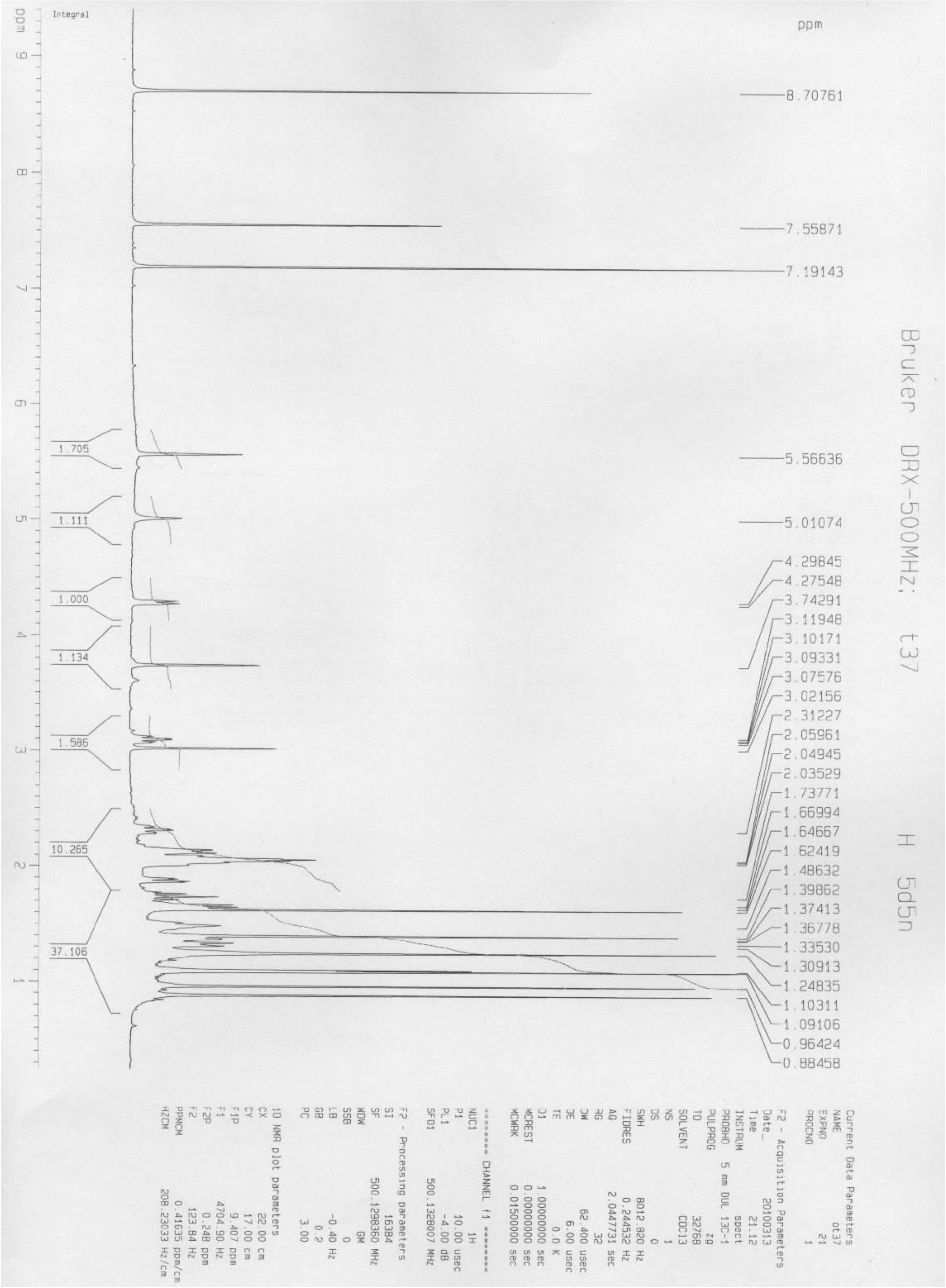

Figure S11. ^{13}C-NMR spectrum (125 MHz) of Euscaphic acid (4) in C_5D_5N.

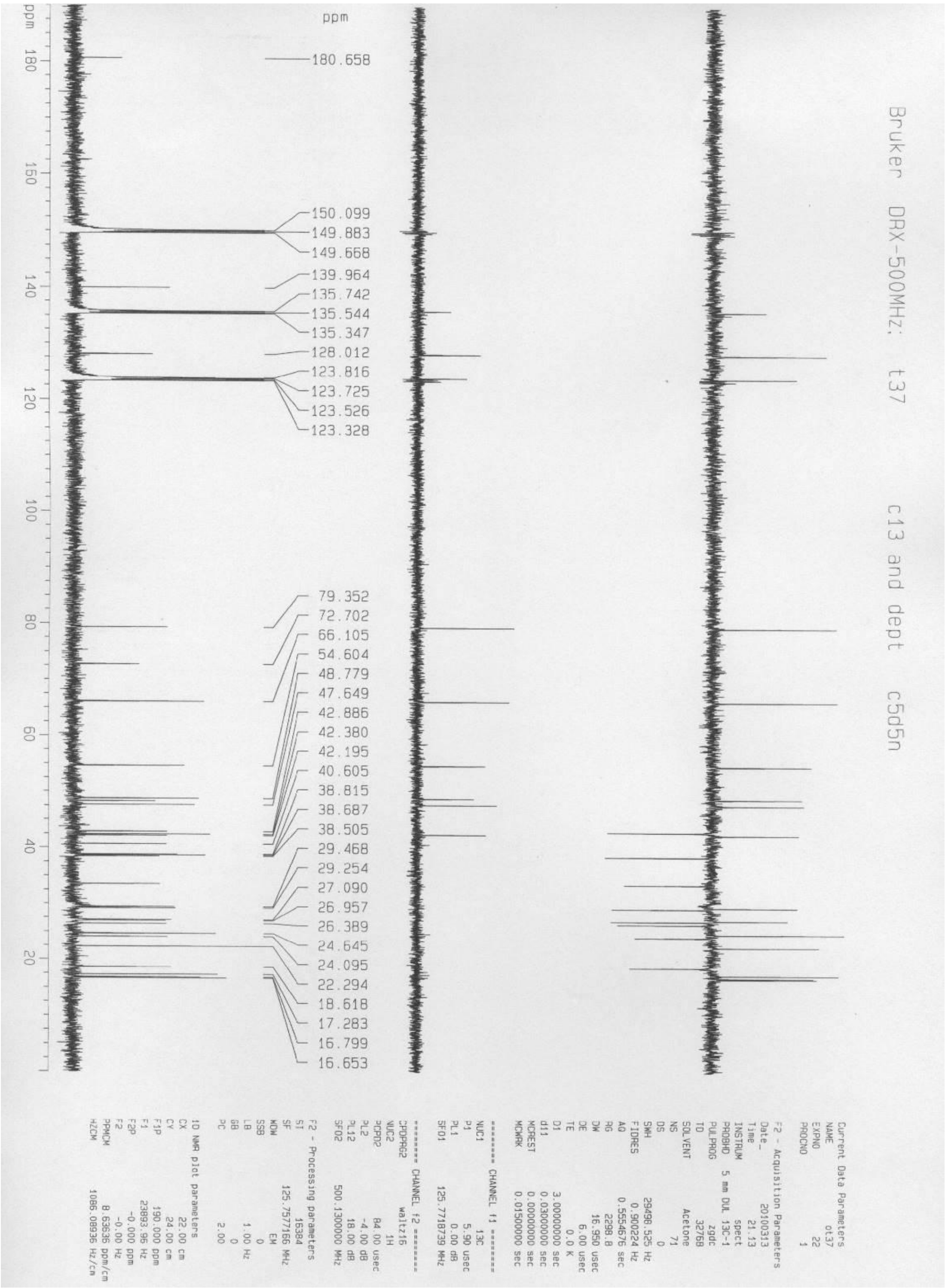

Figure S12. ^{1}H-NMR spectrum (500 MHz) of Myrianthic acid (5) in C_5D_5N.

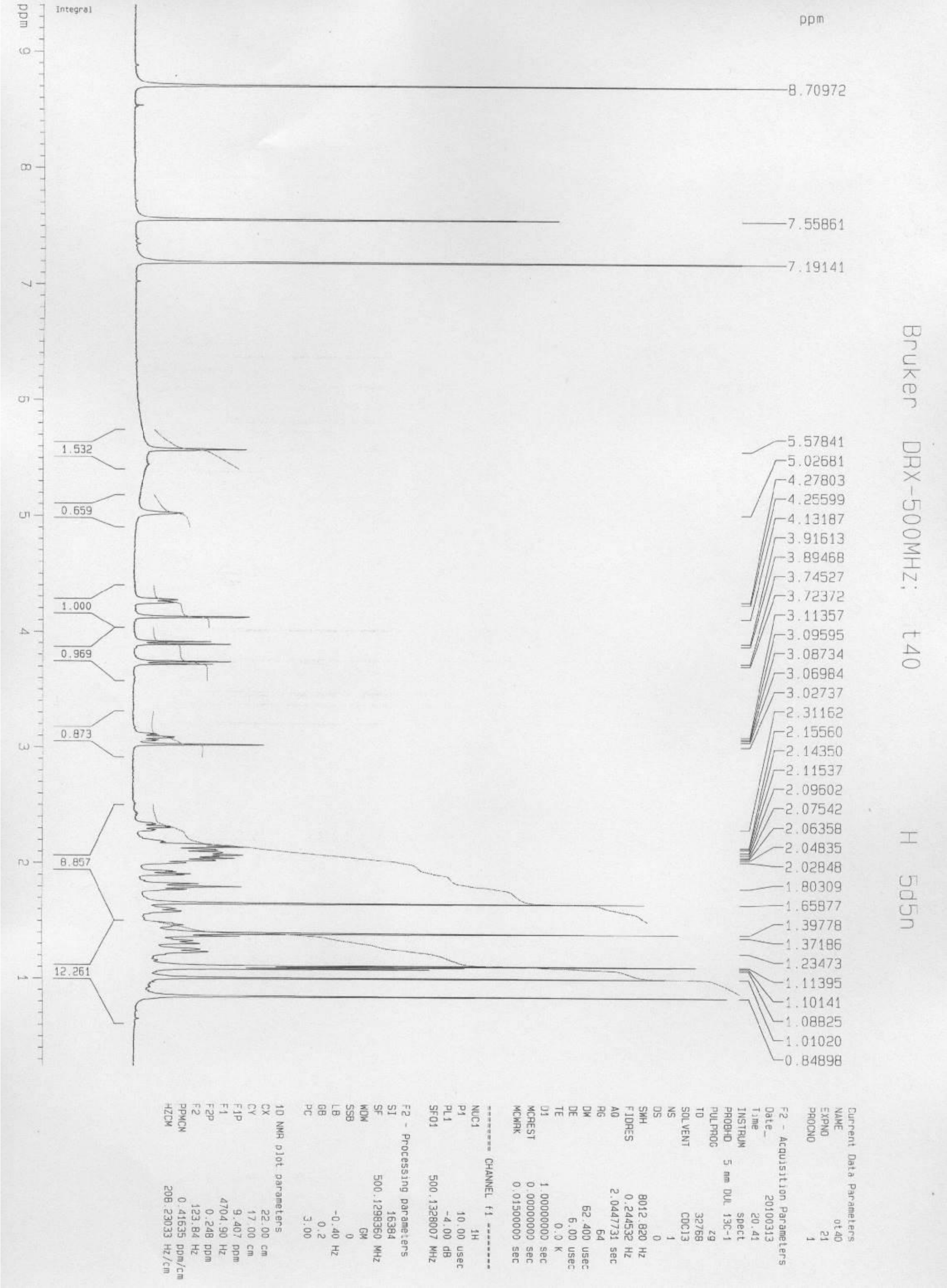

Figure S13. ^{13}C-NMR spectrum (125 MHz) of Myrianthic acid (5) in C_5D_5N.

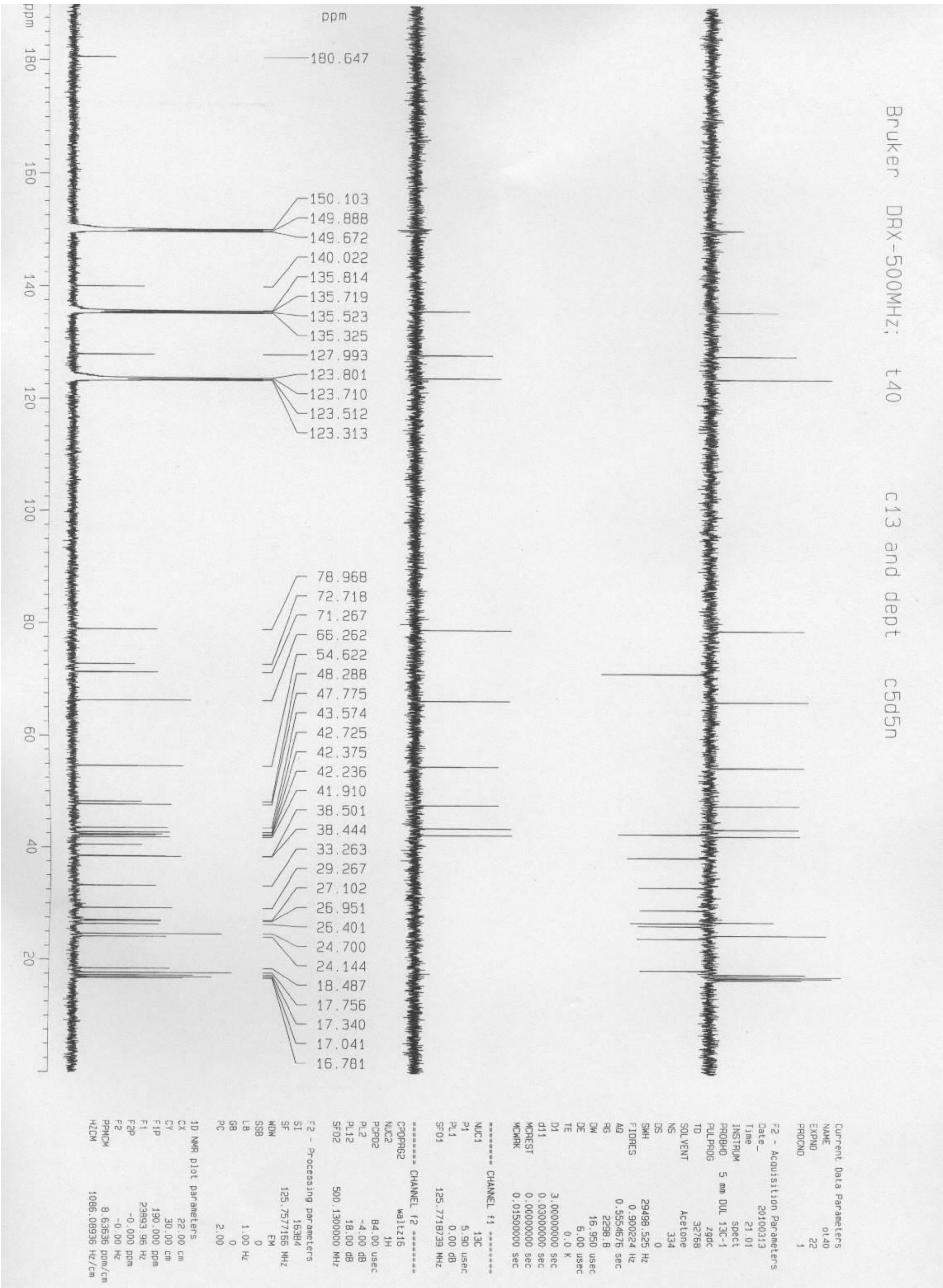

Figure S14. ^{1}H-NMR spectrum (400 MHz) of Lupeol (6) in $CDCl_3$ and CD_3OD (10:1).

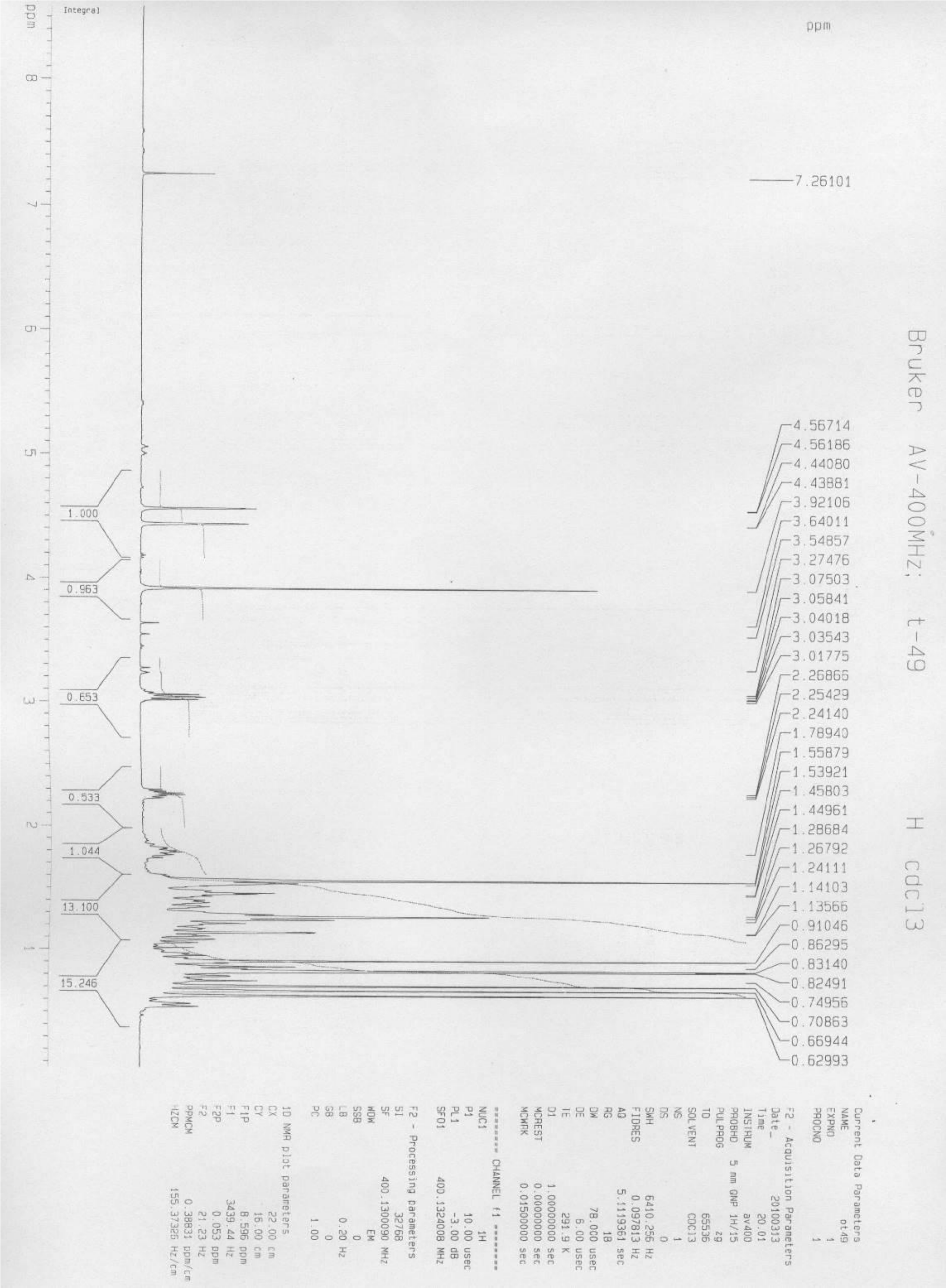

Figure S15. ^{13}C-NMR spectrum (100 MHz) of Lupeol (6) in $CDCl_3$ and CD_3OD (10:1).

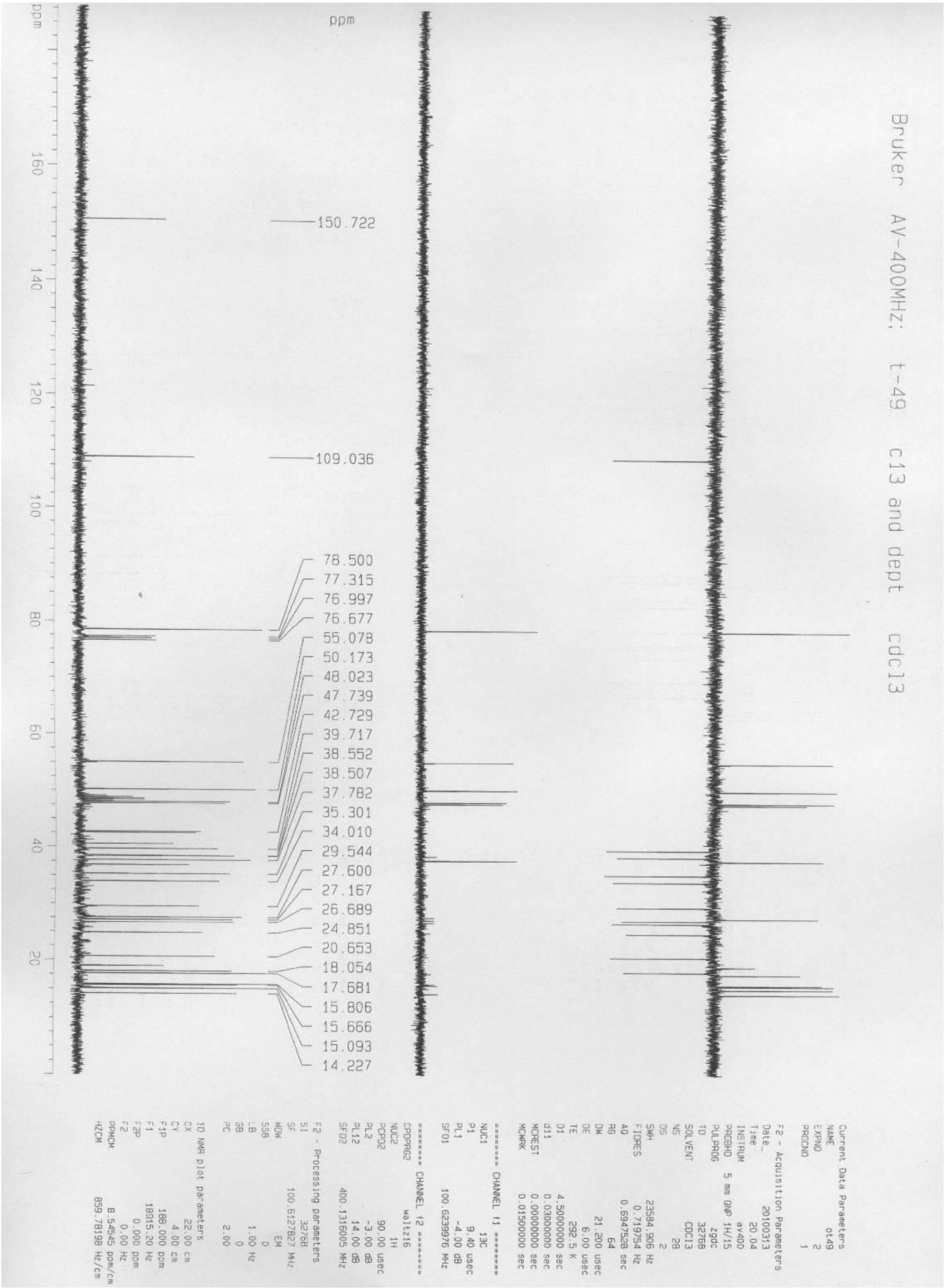

Figure S16. ^{1}H-NMR spectrum (400 MHz) of Betulin (7) in $CDCl_3$ and CD_3OD (10:1).

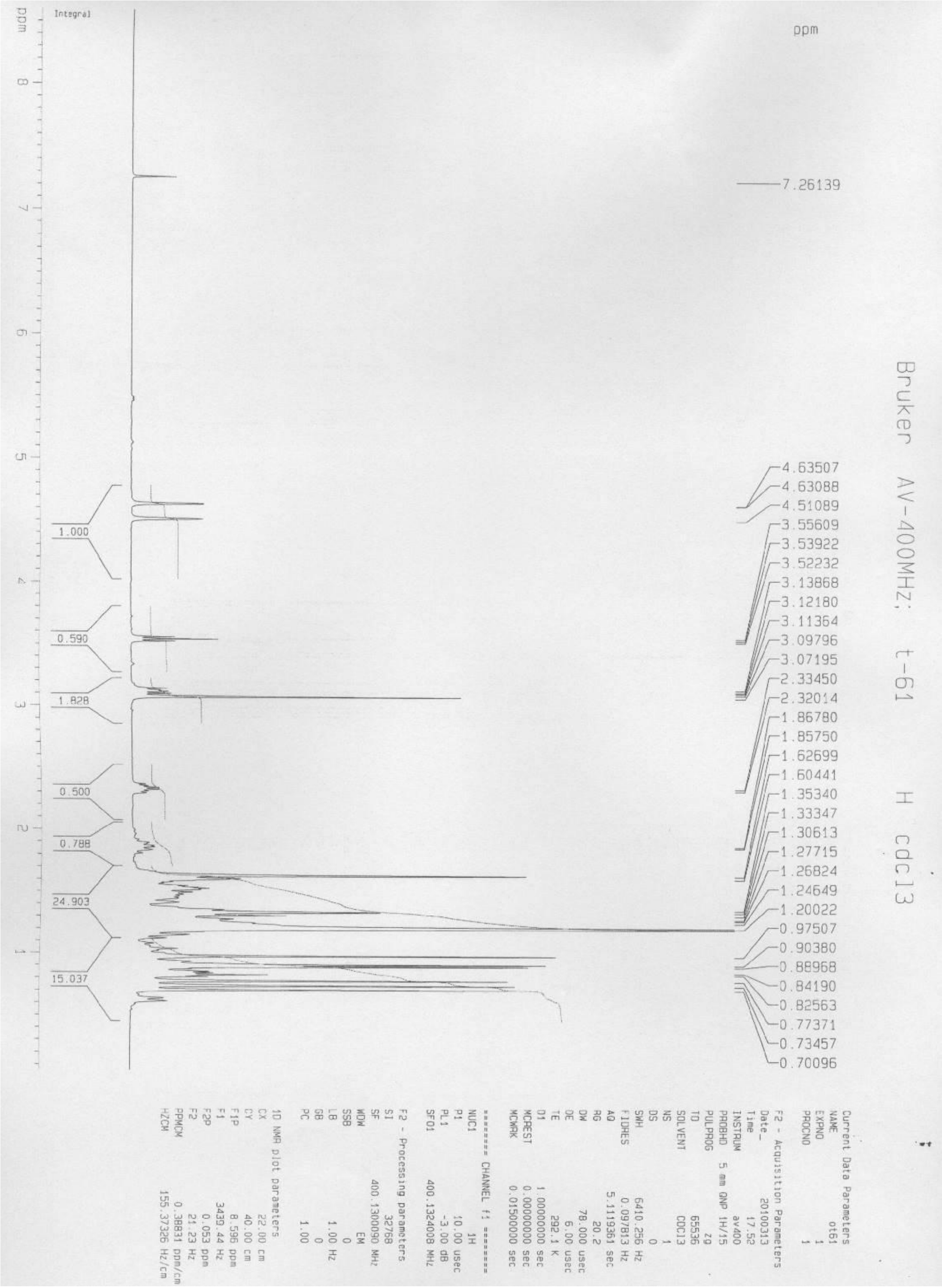

Figure S17. ^{13}C-NMR spectrum (100 MHz) of Betulin (7) in $CDCl_3$ and CD_3OD (10:1).

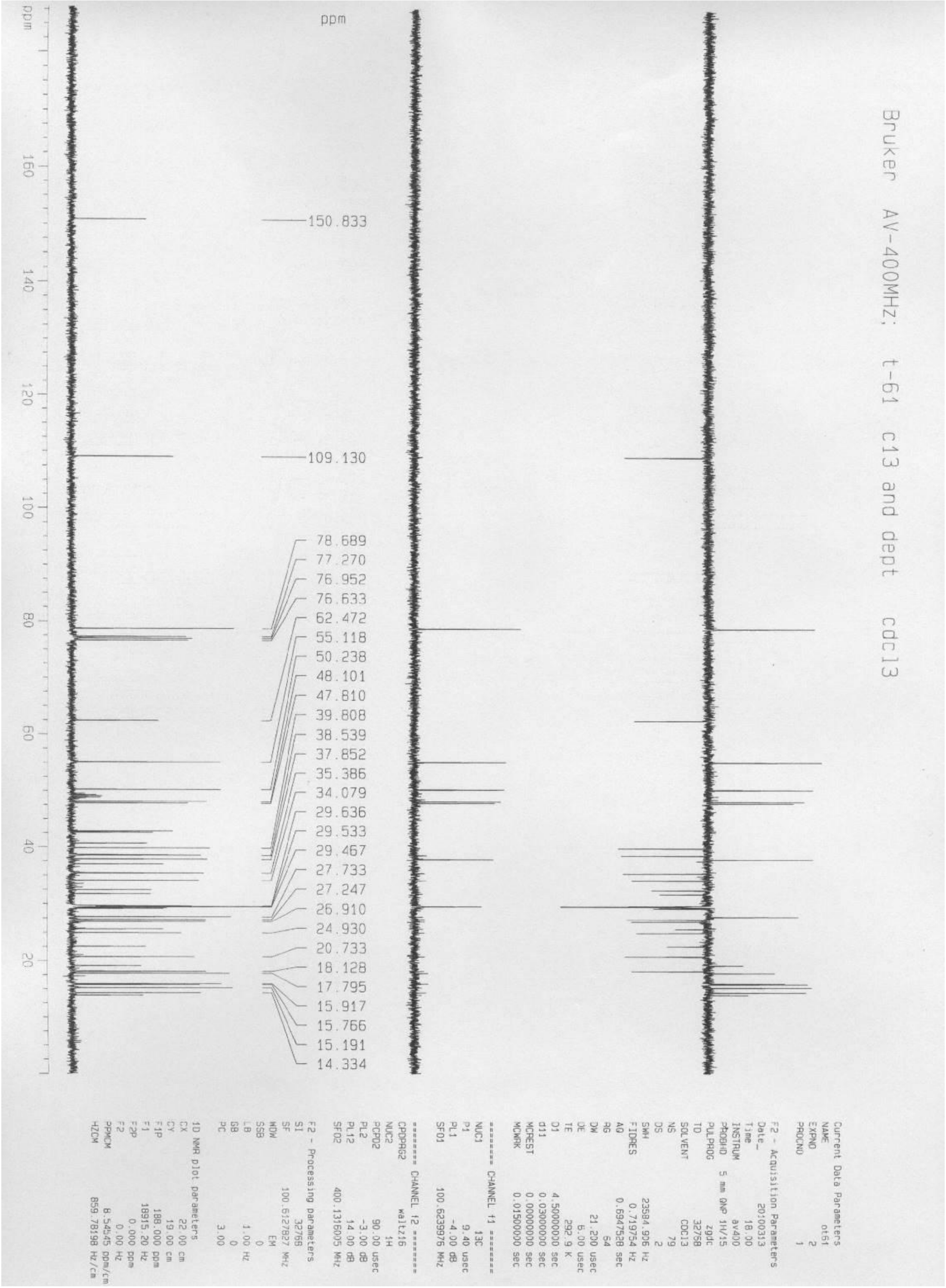

Figure S18. ^{1}H-NMR spectrum (400 MHz) of Betulinic acid (8) in $CDCl_3$ and CD_3OD (10:1).

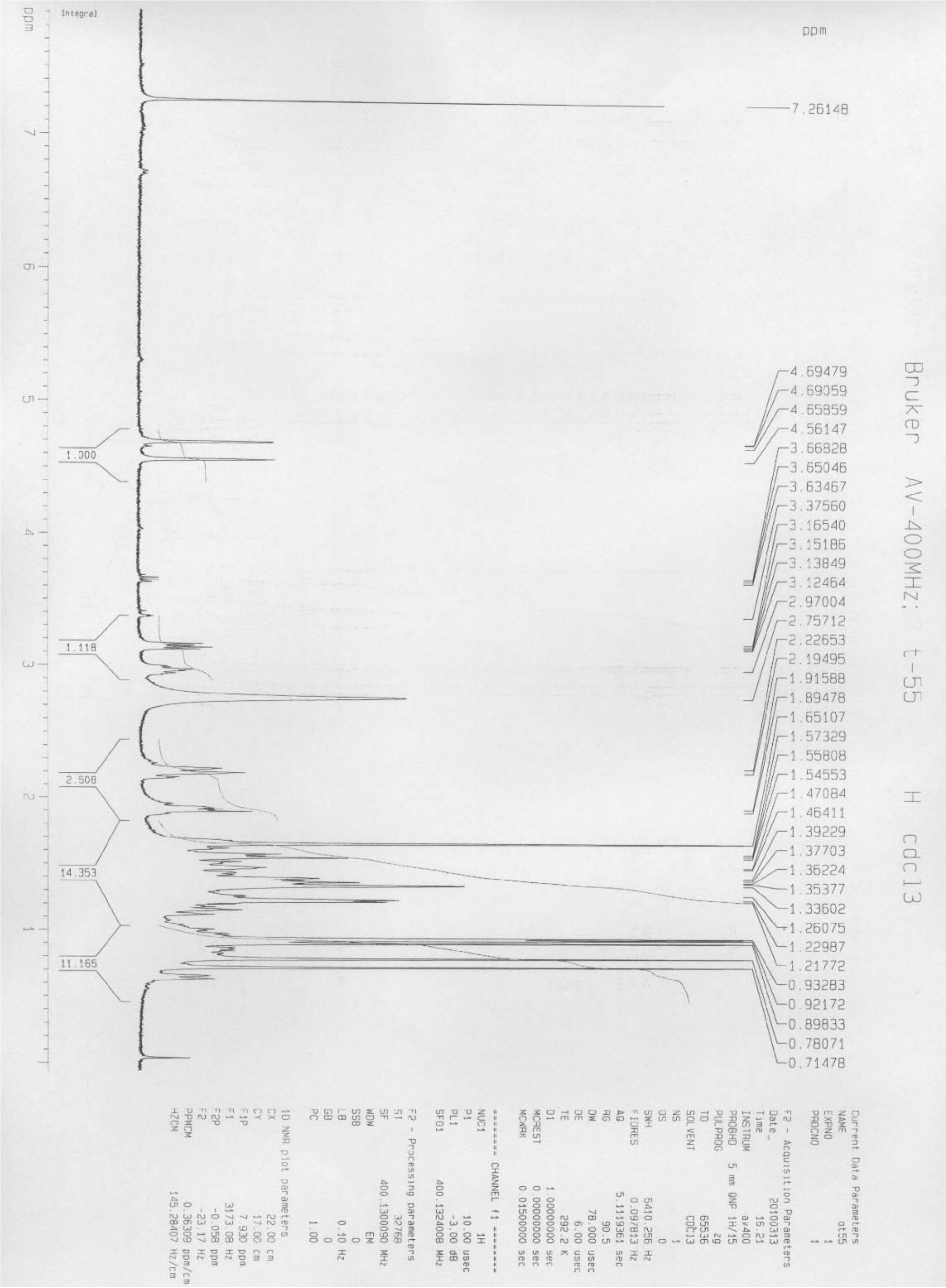

Figure S19. ^{13}C-NMR spectrum (100 MHz) of Betulinic acid (8) in $CDCl_3$ and CD_3OD (10:1).

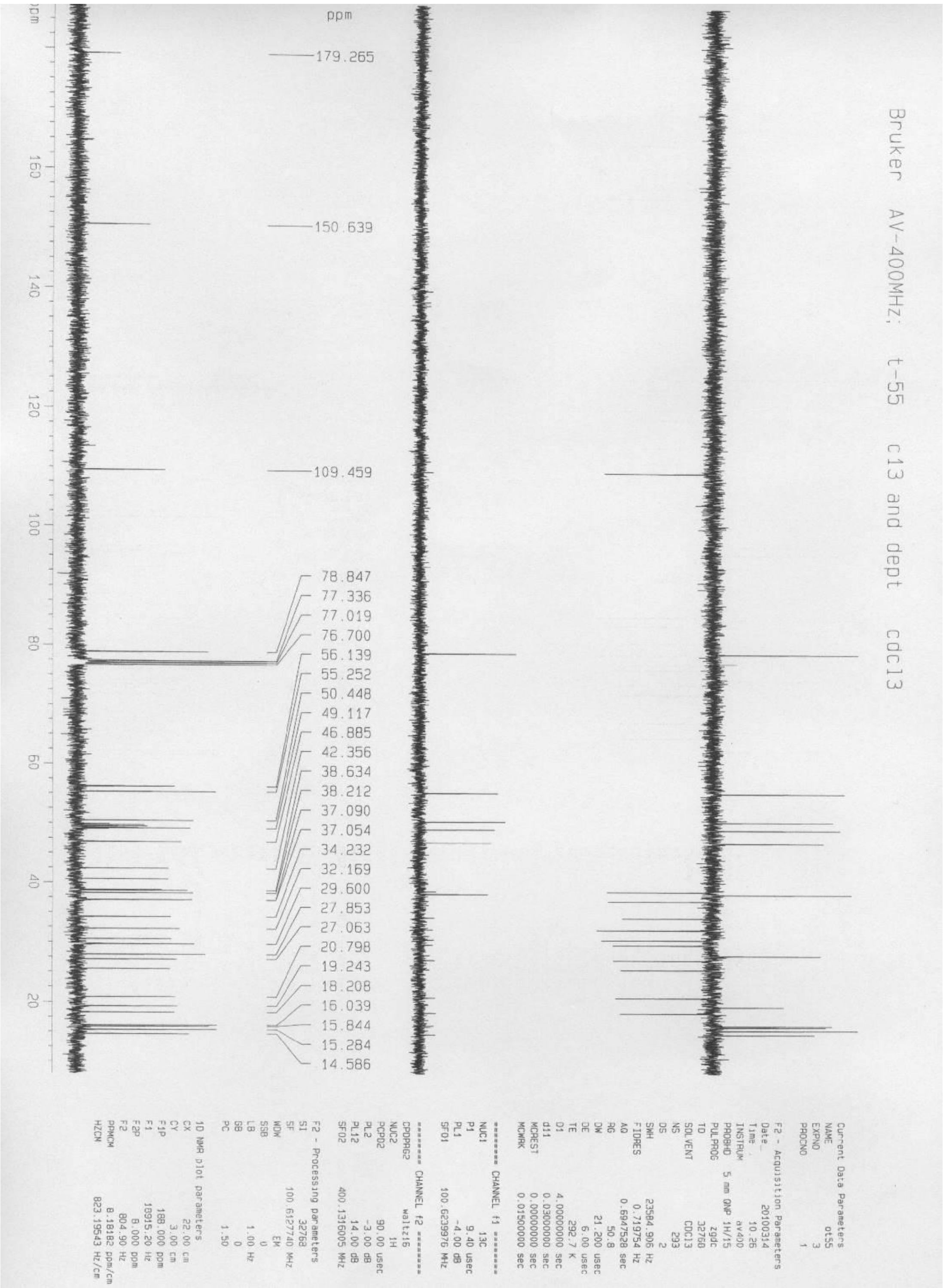

Figure S20. ^{1}H-NMR spectrum (500 MHz) of Plantanic acid (9) in $CDCl_3$ and CD_3OD (10:1).

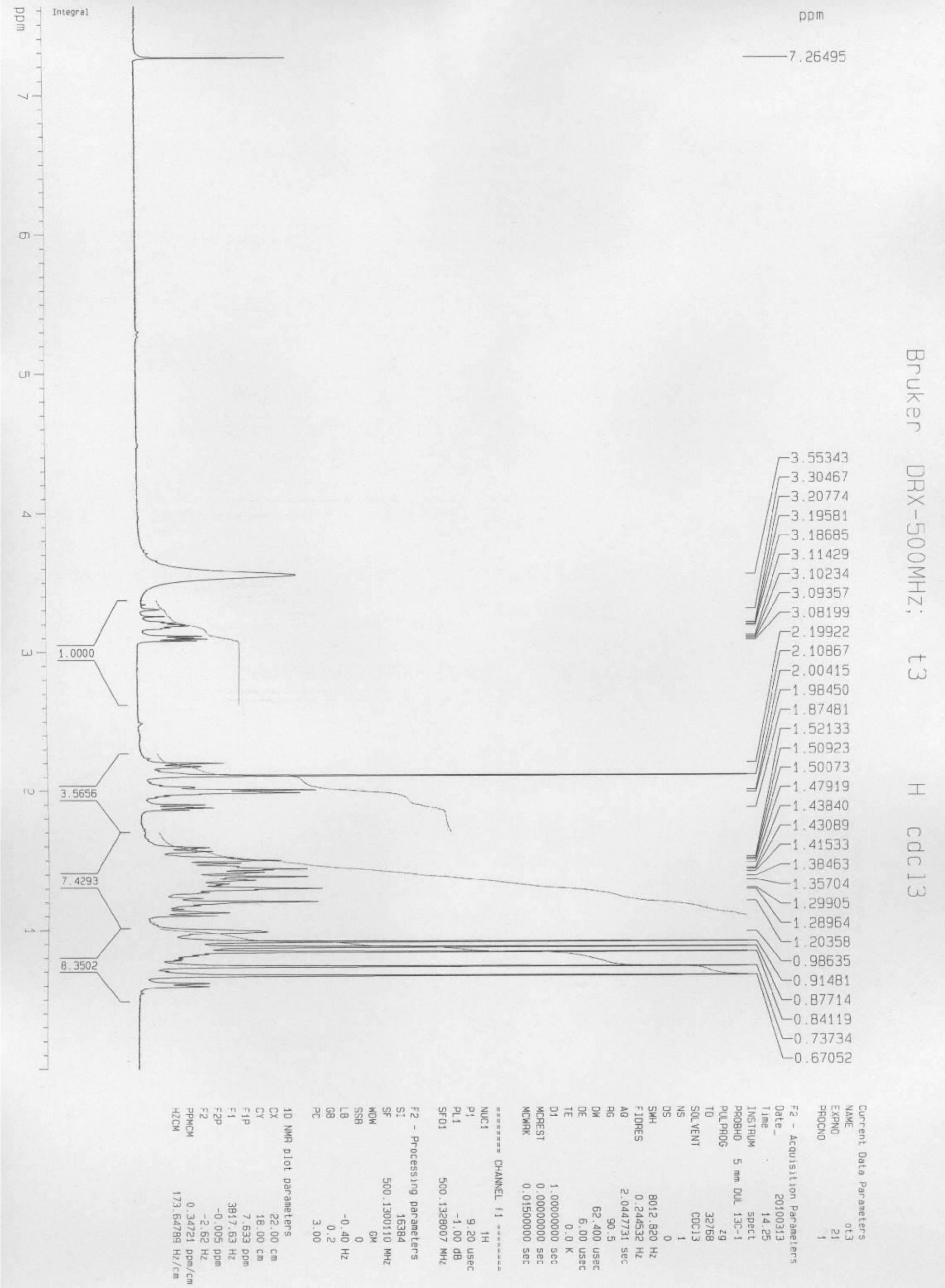

Figure S21. ^{13}C-NMR spectrum (125 MHz) of Plantanic acid (9) in $CDCl_3$ and CD_3OD (10:1).

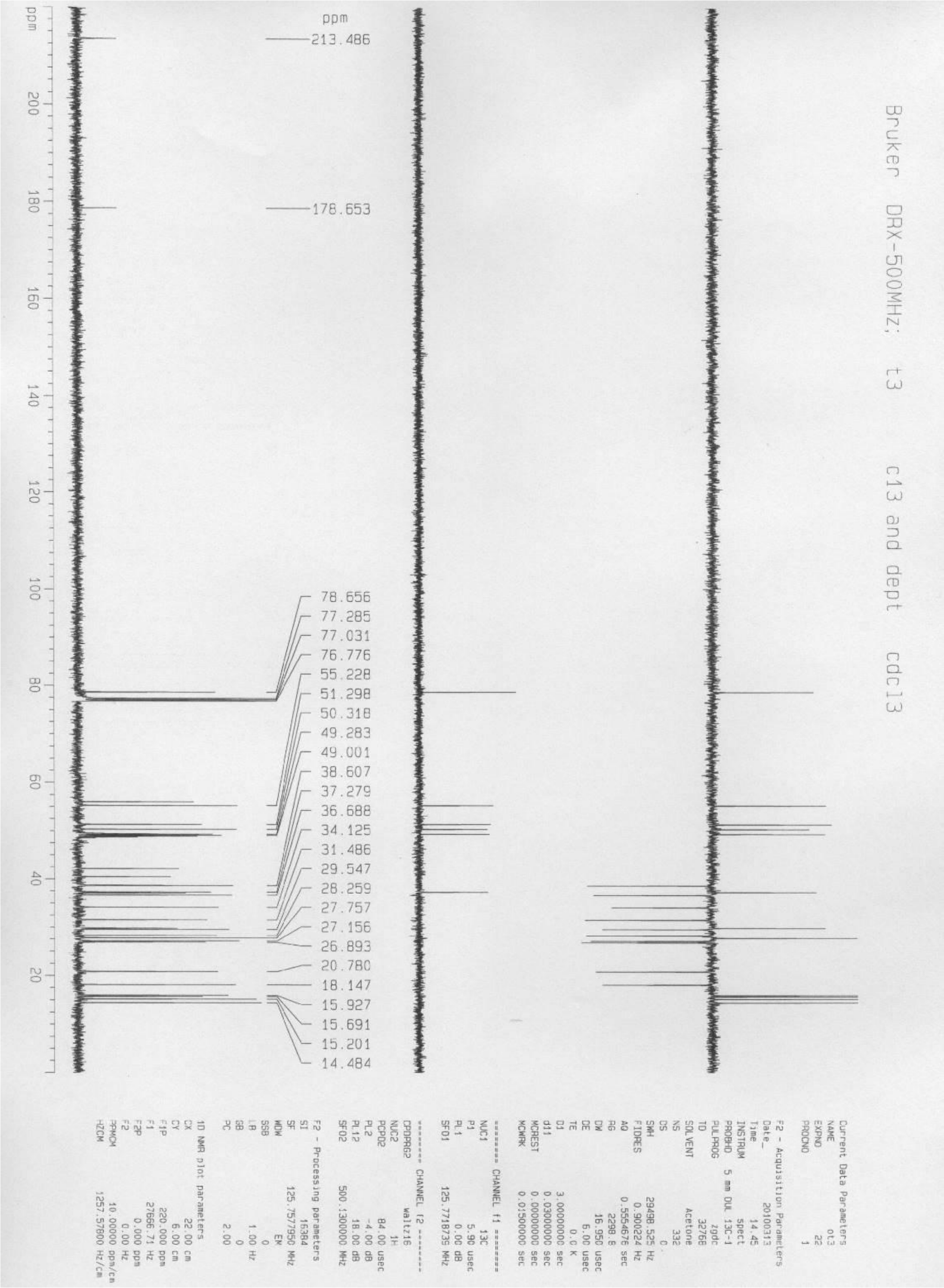

Supplement: Supplementary file 1 [file molecules-19-04897-s001.pdf]
